# Structural enzymology of a *Fusarium graminearum* aldehyde oxidase reveals a distinct active-site and reactivity versus its paralog galactose oxidase

**DOI:** 10.1042/BCJ20260010

**Published:** 2026-03-26

**Authors:** Jessica K. Fong, Laura Mazo, Alison K. Nairn, Rosa Lorizolla Cordeiro, Yann Mathieu, Yu Seby Chen, Carme Rovira, Paul H. Walton, Filip Van Petegem, Harry Brumer

**Affiliations:** 1 Michael Smith Laboratories, University of ­British Columbia, Vancouver BC, Canada; 2 Department of Chemistry, University of British Columbia, Vancouver BC, Canada; 3 Department of Inorganic and Organic Chemistry and Institute of Theoretical and Computational Chemistry (IQTCUB), University of Barcelona, Barcelona, Spain; 4 Department of Chemistry, University of York, York, U.K.; 5 Department of Biochemistry and Molecular Biology, University of British Columbia, Vancouver BC, Canada; 6 Catalan Institution for Research and Advanced Studies (ICREA), Barcelona, Spain

**Keywords:** aldehyde oxidase, Copper Radical Oxidase, Fusarium, galactose oxidase, glyoxal oxidase, methylglyoxal

## Abstract

Copper radical oxidases (CROs), which comprise Auxiliary Activity Family 5 (AA5) in the Carbohydrate-Active Enzymes (CAZy) classification, have a long history of study due to their unique catalytic mechanism and biotechnological applications. The majority of mechanistic and structural insights into CRO function have been obtained from studies on the galactose 6-oxidase from the fungal phytopathogen *Fusarium graminearum* (*Fgr*GalOx) of AA5 subfamily 2 (AA5_2). In contrast, enzyme structure/function studies of CROs from subfamily 1, comprising glyoxal oxidases, are limited. Here, we report the biochemical characterisation of the individual AA5_1 members from *F. graminearum* and *Colletotrichum graminicola*, which exhibit predominant activities on aldehydes, such as methylglyoxal, and enantioselectivity for d-glyceraldehyde. Electron paramagnetic resonance indicated that the AA5_1 aldehyde oxidases possessed similar copper coordination geometry to AA5_2 CROs, including a canonical cross-linked Tyr-Cys residue. However, the X-ray crystal structure of the *F. graminearum* aldehyde oxidase—the first of a fungal AA5_1 CRO—strikingly revealed that a key radical-stabilising tryptophan side chain in the second coordination sphere is provided by a different position in the polypeptide chain and exists in a flipped orientation vis-à-vis AA5_2 members. Quantum mechanics/molecular mechanics (QM/MM) calculations demonstrated that, in contrast to the AA5_2 GalOx, the AA5_1 aldehyde oxidase does not delocalise spin density onto the second-sphere tryptophan as a consequence of this alternative active-site arrangement. Together, these data provide new molecular insight into catalytic selectivity among the distinct subfamilies of alcohol- and aldehyde-specific CROs, which will facilitate elucidation of their biological roles and inform their application as biocatalysts.

## Introduction

Copper radical oxidases (CROs) are mononuclear metalloenzymes that catalyse the two-electron oxidation of primary alcohols and aldehydes to their corresponding aldehydes and carboxylic acids, respectively. Molecular oxygen is reduced to hydrogen peroxide as a co-product. Unique among oxidoreductases, CROs employ a protein-derived cofactor comprised of a cross-linked cysteine-tyrosyl radical, which is electronically coupled with the copper centre [1–7]. CROs were first isolated from ascomycete fungi as early as the 1950s and are also now known to be produced by bacteria, plants, and insects [7]. All CROs are classified by protein sequence into CAZy Auxiliary Activity Family 5 (AA5), with fungal sequences further segregated into Subfamily 1 (AA5_1, comprising glyoxal oxidases) and Subfamily 2 (AA5_2, comprising galactose-6-oxidases and other primary alcohol oxidases) [7–10].

The archetypal CRO is the galactose 6-oxidase (GalOx, EC 1.1.3.9) that was first isolated in 1959 from the wheat blight pathogen *Fusarium graminearum* [11]. Since its discovery, *Fgr*GalOx has been the subject of extensive mechanistic and structural studies [2,12–23]. Moreover, its distinct chemistry has spurred diverse biocatalysis applications, ranging from polysaccharide modification to pharmaceutical synthesis [24–31] (for additional examples, see reviews [6,7]). Recent exploration of the sequence diversity within AA5_2 has revealed that this subfamily also contains a range of general alcohol oxidases (AlcOx, EC 1.1.3.13) and aryl alcohol oxidases (AAOs, EC 1.1.3.7), in addition to specific galactose-6-oxidases [32–37]. Crystal structures have been solved for CROs from each of these specificity classes, i.e., *Fgr*GalOx [12], *Cgr*AlcOx [32], and *Cgr*AAO [34], which have enabled comparative structural enzymology within AA5_2.

The primary representative of AA5_1 is the glyoxal oxidase (GlyOx, EC 1.2.3.15) from the white-rot fungus *Phanerochaete chrysosporium*, which was first isolated in 1987, based on an association with lignocellulose degradation [38,39]. Despite its name, this enzyme is poorly specific for glyoxal (and equally so on a range of small aldehydes) and exhibits predominant activity on methylglyoxal [38]. Detailed biochemical studies on *Pch*GlyOx led to the suggestion that this enzyme oxidises aldehydes via the hydrated *gem*-diol form, thus demonstrating mechanistic analogy to alcohol oxidation by AA5_2 CROs [40]. Several additional ‘glyoxal’ (aldehyde) oxidases have now been characterised from AA5_1, some of which have been implicated in fungal and bacterial morphogenesis [41–46]. Like AA5_2 CROs, the distinct catalytic properties of these enzymes have motivated biotechnological applications [47–51].

Despite this considerable, sustained interest in the biochemistry, biology, and biotechnology of CROs, structure-activity analyses of these enzymes have been limited by the existence of comparatively few experimental tertiary structures [12,32,34,46]. In particular, no AA5_1 structures have been solved previously (see https://www.cazy.org/AA5_structure.html). As introduced above, *F. graminearum* was the source for the seminal AA5 GalOx structure (subfamily 2) [12]. We therefore selected the *F. graminearum* AA5_1 paralog to address this knowledge gap, also with an eye towards completing the biochemical and structural characterisation of the CROs in this important fungal phytopathogen. We demonstrate here, through enzyme kinetics and product analysis, that the *F. graminearum* AA5_1 member is a *bona fide* aldehyde oxidase, with predominant activities on d-glyceraldehyde and methylglyoxal (and very limited activity of glyoxal). We also present the experimental crystal structure of this enzyme as the first of an AA5_1 member, together with electron paramagnetic resonance (EPR) and QM/MM analysis of the active site. In addition, the biochemical characterisation of an ortholog from *Colletotrichum graminicola* highlighted the catalytic similarity of AA5_1 CROs between two important phytopathogenic fungi.

## Results and discussion

### Target selection and bioinformatic analysis

The genome of *F. graminearum* (https://www.cazy.org/e3987.html) encodes four AA5_2 members (Genbank: CEF87161, CEF78396, CEF72141, and CEF83807) and a single AA5_1 member (CEF86606). Of these, only two AA5_2 members have been biochemically characterised: *Fgr*GalOx (CEF87161), the galactose 6-oxidase archetype [12], and *Fgr*AAO (CEF78396), an aryl alcohol oxidase [36]. As introduced above, *Fgr*GalOx was the first tertiary structural representative of the subfamily [12]. Similarly, the genome of *Colletotrichum graminicola* (https://www.cazy.org/e29653.html) encodes three AA5_2 members (EFQ36699, EFQ30446, and EQF27661) and a single AA5_1 member (EFQ26204). Of these, the three AA5_2 members have been individually characterised as a general alcohol oxidase (*Cgr*AlcOx, EFQ30446) [32], a GalOx/raffinose oxidase (*Cgr*RafOx, EFQ36699) [33], and an aryl alcohol oxidase (*Cgr*AAO, EFQ27661) [34]. Tertiary structures have been solved for *Cgr*AlcOx [32] and *Cgr*AAO [34]. Thus, given the relative paucity of information available on AA5_1 members, we selected the corresponding AA5_1 orthologs from *F. graminearum* and *C. graminicola* for biochemical and structural characterisation. Hereafter, these proteins are denoted as *Fgr*AldOx and *Cgr*AldOx, respectively, in consideration of their flexible substrate scope as aldehyde oxidases (EC 1.2.3.-; *vide infra*).

Protein sequence analysis indicated the presence of three N-terminal ‘wall stress-responsive component’ (WSC) domains and a C-terminal AA5_1 catalytic module in both *Fgr*AldOx and *Cgr*AldOx. WSC domains are identified via their characteristic PAN/Apple domain fold and four conserved disulphide bridges (eight cysteine residues; Supplementary Figure S1). Phylogenetic analysis, based on the AA5 catalytic modules alone, revealed that AA5_1 members with WSC domains separate into a monophyletic group that diverges early from those that lack WSC domains (Supplementary Figure S2).

Multiple sequence alignment of the isolated AA5_1 and AA5_2 catalytic modules revealed that the *Fgr*AldOx catalytic module has low sequence identities of 17% and 18%, respectively, with its AA5_2 paralogs, *Fgr*GalOx [12] and *Fgr*AAO [36] (Supplementary Tables S1 and S2). Similarly, *Cgr*AldOx shares 16%–19% sequence identities with its AA5_2 paralogs, *Cgr*AlcOx, *Cgr*RafOx, and *Cgr*AAO [32–34]. While *Fgr*AldOx and *Cgr*AldOx share 51% identity, a distantly related bacterial glyoxal oxidase, GlxA from *Streptomyces lividans*, shares only a 16% sequence identity with *Fgr*AldOx or *Cgr*AldOx [45]. Despite these low sequence identities, all first-shell active site residues are directly conserved in *Fgr*AldOx and *Cgr*AldOx and across AA5_1 and AA5_2 (Supplementary Figure S3). Notably, an aromatic residue in the second coordination sphere of AA5_2 enzymes (W290 in *Fgr*GalOx, F138 in *Cgr*AlcOx, Y334 in *Cgr*AAO), which stacks with the cross-linked Tyr-Cys cofactor and is important for activity [32,34,52], lacks an obvious homolog in AA5_1 members, based on sequence alignment (Supplementary Figure S3). The implications of this are discussed further in light of the *Fgr*AldOx crystal structure presented below.

### Recombinant protein production

The commercial synthesis of cDNA corresponding to the full-length AA5_1 sequences from *F. graminearum* and *C. graminicola* was unsuccessful. However, cDNA sequences comprising only the catalytic module, or with a single WSC domain, were obtained. The synthetic genes also included a C-terminal hexahistidine tag. Recombinant expression of constructs comprising only the catalytic module was unsuccessful in *Komagataella phaffii* (syn. *Pichia pastoris*) X-33. On the other hand, constructs containing one WSC domain were successfully expressed to yield secreted, stable proteins. Yields of purified *Fgr*AldOx and *Cgr*AldOx were typically 6 and 1 mg per litre of culture, respectively. SDS-PAGE analysis of both recombinant proteins indicated higher molecular weights than expected for the WSC-AA5_1 constructs. Deglycosylation with PNGase F confirmed the presence of N-glycosylation with shifts in electrophoretic mobility corresponding to 20 kDa for *Fgr*AldOx and 14 kDa for *Cgr*AldOx (Supplementary Figure S4).

ICP-MS was used to determine the copper content of both enzymes. Analysis of initial samples indicated a copper-to-protein molar ratio greater than 1:1 (up to 3:1 in some preparations). Therefore, samples were treated with a low concentration of EDTA (5 mM) followed by buffer exchange, resulting in final copper-to-protein ratios of 1.2 ± 0.2 for *Fgr*AldOx and 1.0 ± 0.1 for *Cgr*AldOx, indicating that the proteins were fully loaded with copper. Enzyme activities on methylglyoxal (*vide infra*) of both *Fgr*AldOx and *Cgr*AldOx before and after EDTA treatment were comparable, indicating that excess copper did not affect kinetics.

### Substrate specificities

WSC-containing AA5 enzymes have been reported to bind polysaccharides such as chitin and laminarin [43,53]. To investigate the potential carbohydrate-binding abilities of *Fgr*AldOx and *Cgr*AldOx, affinity gel electrophoresis was performed using several polysaccharides (laminarin, xylan, galactomannan, shrimp chitin, arabinogalactan, and xyloglucan). However, no binding was observed (Supplementary Figure S5).

**Figure 1:**
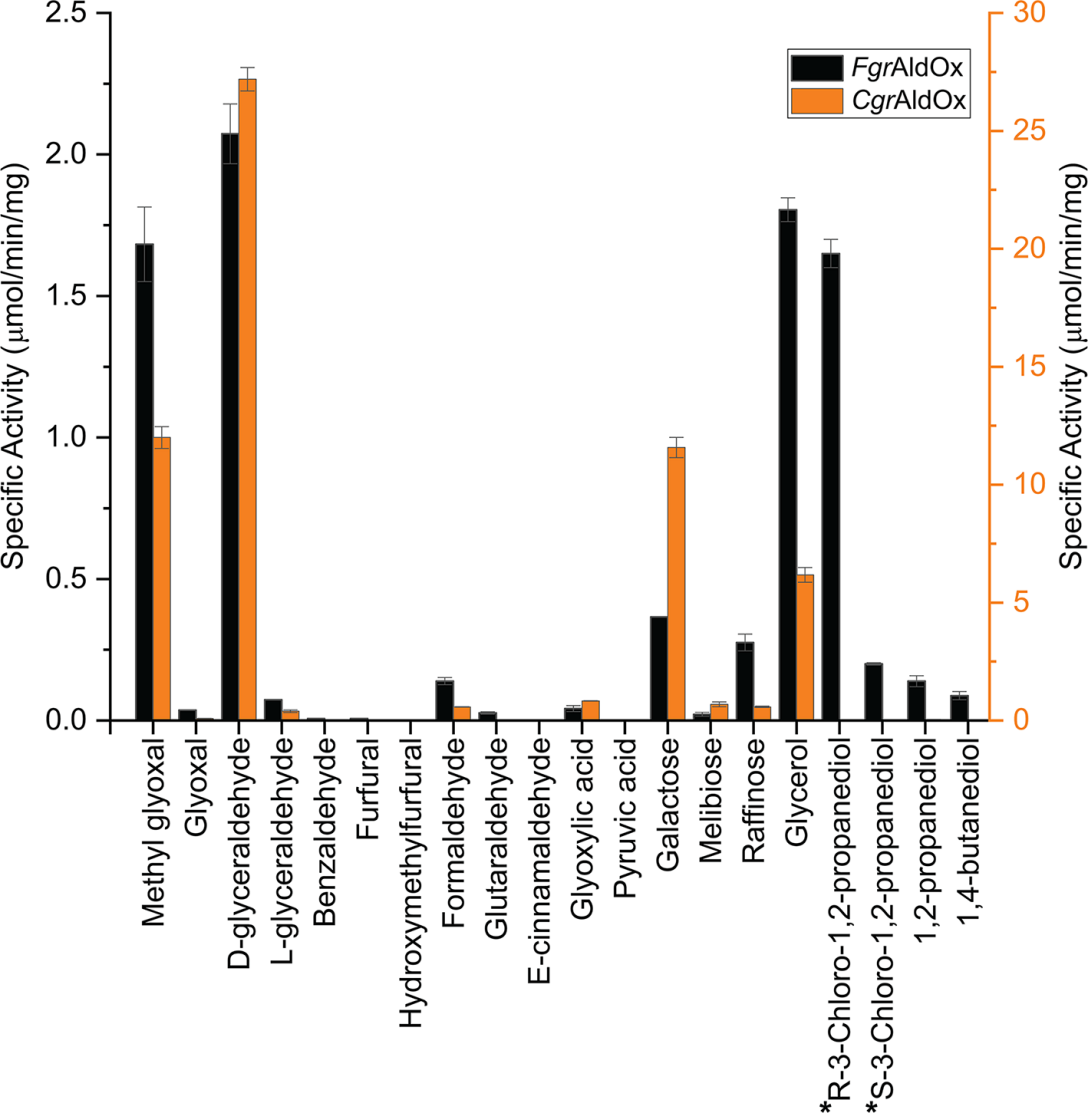
Activity profiles of *Fgr*AldOx and *Cgr*AldOx activity on a representative substrate set Individual values are presented in Supplementary Table S3. Specific activity was determined using the HRP-ABTS-coupled assay with 50 mM sodium phosphate buffer, pH 7.0, at ambient temperature. Measurements were performed in triplicate with 10 mM alcohols and aldehydes, except for carbohydrates, glycerol, and *R*- and *S*-3-chloro-1,2-propanediols (* not tested with *Cgr*AldOx), which were tested at 300 mM. Note the distinct *y*-axes for each enzyme.

Activity screening was first carried out on a small panel of typical CRO substrates at room temperature and pH 7.0 (Figure 1 and Supplementary Table S3). Methylglyoxal, the predominant substrate for most AA5_1 members [38,41,42,48,50] was subsequently chosen to determine pH-rate and temperature stability profiles. The pH optima of *Fgr*AldOx and *Cgr*AldOx were pH 8.0 and 6.0, respectively (Supplementary Figure S6). Stability assays over the course of 2 days indicated that both *Fgr*AldOx and *Cgr*AldOx were most stable at 30–35°C (Supplementary Figure S7).

The specific activity profiles of *Fgr*AldOx and *Cgr*AldOx were highly similar, although *Cgr*AldOx was considerably (4- to 20-fold) more active on the same substrates (Figure 1 and Table 1). High specific activity was observed for both enzymes on methylglyoxal, yet specific activity on glyoxal was very low. This disparity has been observed previously with other characterised AA5_1 CROs, including the first example from *P. chrysosporium*, yet these enzymes are often called ‘glyoxal’ oxidases [38,42,44,48,50]. Product analysis of methylglyoxal oxidation confirmed the product of *Fgr*AldOx was pyruvic acid, with 75% conversion of a 40 mM aqueous solution after 12 h using an enzyme load of 7 μM (Supplementary Figure S8). Notably, the highest specific activities of *Fgr*AldOx and *Cgr*AldOx were observed on d-glyceraldehyde (Figure 1). Both enzymes poorly oxidised the l-isomer, indicating high stereoselectivity. Interestingly, *Fgr*AldOx and *Cgr*AldOx were essentially inactive on aryl aldehydes, including benzaldehyde, cinnamaldehyde, furfural, and 5-hydroxymethylfurfural, the last two of which are of particular interest as biomass-derived platform chemicals [36,51,54,55].

**Table 1:** Michaelis–Menten kinetic parameters for selected substrates

Enzyme	Substrate	*K_M_* (mM)	*k*_cat_ (s^−1^)	*k*_cat_/*K*_M_ (M^−1^s^−1^)	pH	Temperature (°C)
*Fgr*AldOx	d-glyceraldehyde	233 ± 36	129 ± 17	550 (500)^a^	8.0	30
	Methylglyoxal	22 ± 4	7.4 ± 0.6	330 (210)^a^		
	Glyoxal	237 ± 20	1.5 ± 0.1	6 (6)^a^		
	Formaldehyde	n.a.	n.a.	(13)^a^		
	Glycerol	n.a.	n.a.	(9)^a^		
	Galactose	n.a.	n.a.	(2)^a^		
	Raffinose	483 ± 36	(8.2 ± 0.4) × 10^−1^	2 (2)^a^		
*Cgr*AldOx	d-glyceraldehyde	31 ± 2	110 ± 6	3550 (2750)^a^	6.0	35
	Methylglyoxal	7.4 ± 2.5 n.a. (37 ± 9)^b^	13.0 ± 1.4 n.a. (43 ± 9)^b^	1755 (995)^a^ (1160)^b^		
	Glycerol	1870 ± 340	52 ± 8	28 (27)^a^		
	Galactose	n.a.	n.a.	(35)^a^		
	Melibiose	106 ± 18	(4.2 ± 0.3) × 10^−1^	4 (2)^a^		

a Values in parentheses correspond to *k*_cat_/*K*_M_ values determined from the slopes of linear fits to initial-rate kinetic data substrate concentrations well below saturation; individual *K*_M_ and *k*_cat_ values were not calculated. n.a. = not applicable.

b Kinetic parameters were obtained by fitting a modified Michaelis–Menten equation including a term for substrate inhibition

*Fgr*AldOx and *Cgr*AldOx demonstrated low or undetectable activity on aryl alcohols and alkane diols (Figure 1). Notably, however, both enzymes had considerable specific activity on the polyols glycerol and d-galactose at high substrate concentrations (Figure 1). Although conversion was poor in the case of d-galactose (110 mM galactose, 7 μM enzyme, 8% conversion after 24 h; Supplementary Figure S9), proton NMR analysis showed that *Fgr*AldOx oxidised the C6-OH of galactose, like *Fgr*GalOx [56].

Determining the stereoselectivity of *Fgr*AldOx for the prochiral molecule glycerol was complicated by the subsequent oxidation of the product glyceraldehyde (*vide supra*). Early work on *Fgr*GalOx demonstrated that this AA5_2 CRO produced predominantly l-glyceraldehyde (*S*-glyceraldehyde) [57] (*S:R*, 96:4) [35]. This specificity was rationalised by superposition with d-galactose, in which glycerol is more favourably orientated in a pro-*S* position for oxidation (Supplementary Figure S10A). Further validation came from the observation of similar activity on *R*-3-chloro-1,2-propanediol, which can be likewise superposed [57] (Supplementary Figure S10A). Inspired by this study, we determined the specific activities of *Fgr*AldOx on *R*- and *S*-3-chloro-1,2-propanediol. The specific activity on the *R*-enantiomer was observed to be comparable to that on glycerol, while the specific activity on the S-enantiomer was *ca*. ten-fold lower (Figure 1). These data suggest, by analogy, that *Fgr*AldOx preferentially oxidises glycerol with a pro-*S* orientation in the active site, like *Fgr*GalOx (Supplementary Figure S10B). However, it should be noted that glycerol is in fact a poor substrate for *Fgr*AldOx, as shown by Michaelis–Menten kinetic analysis; the activity and selectivity for d-(*R*)-glyceraldehyde are far higher (*vide infra*, Table 1).

To further refine the substrate specificity analysis (Figure 1), initial-rate kinetics were obtained for a selection of compounds for *Fgr*AldOx and *Cgr*AldOx. In general, enzyme saturation was difficult to achieve, even for the best substrates (Supplementary Figures S11 and S12). Hence, we used linear fitting of the data at lower substrate concentrations to determine *k*_cat_/*K*_M_ values (*v*_o_/[E]_t_ = *k*_cat_/*K*_M_ [S], where [S] << *K*_M_) and we extracted individual *k*_cat_ and *K*_M_ values by fitting the full Michaelis–Menten equation where possible (Table 1). Mirroring the specific activity data, initial-rate kinetics showed that *Fgr*AldOx and *Cgr*AldOx displayed the highest selectivities for d-glyceraldehyde, with *k*_cat_/*K*_M_ values approximately twice those of methylglyoxal for both enzymes. Focusing on *Fgr*AldOx as an exemplar, glyoxal, which commonly lends its name to AA5_1 CRO, was a poor substrate, with a *k*_cat_/*K*_M_ value 50-fold lower than methylglyoxal (Table 1) and a ca. 10-fold lower initial rate at the highest substrate concentrations tested (70–80 mM; Supplementary Figure S11). Formaldehyde and the alcohols glycerol, galactose, and raffinose likewise exhibited poor selectivities, with comparable *k*_cat_/*K*_M_ values to glyoxal. However, it is notable that glycerol could be tested at concentrations up to 5 M, where the initial-rate kinetics (*v*_o_/[E]_t_) were comparable to d-glyceraldehyde at 50 mM (Supplementary Figures S11 and S12). These data additionally rationalise the relatively high specific activities observed for glycerol versus d-glyceraldehyde shown in Figure 1, where these substrates were assayed at 300 and 10 mM, respectively. Taken together, the kinetic data indicate that both *Fgr*AldOx and *Cgr*AldOx exhibit a strong overall preference for aldehydes and a limited substrate overlap with their AA5_2 paralogs, *Fgr*GalOx [12], *Fgr*AAO [36], *Cgr*AlcOx [32], *Cgr*RafOx [33], and *Cgr*AAO [34].

### Enzyme structural studies

#### Electron paramagnetic resonance

**Figure 2:**
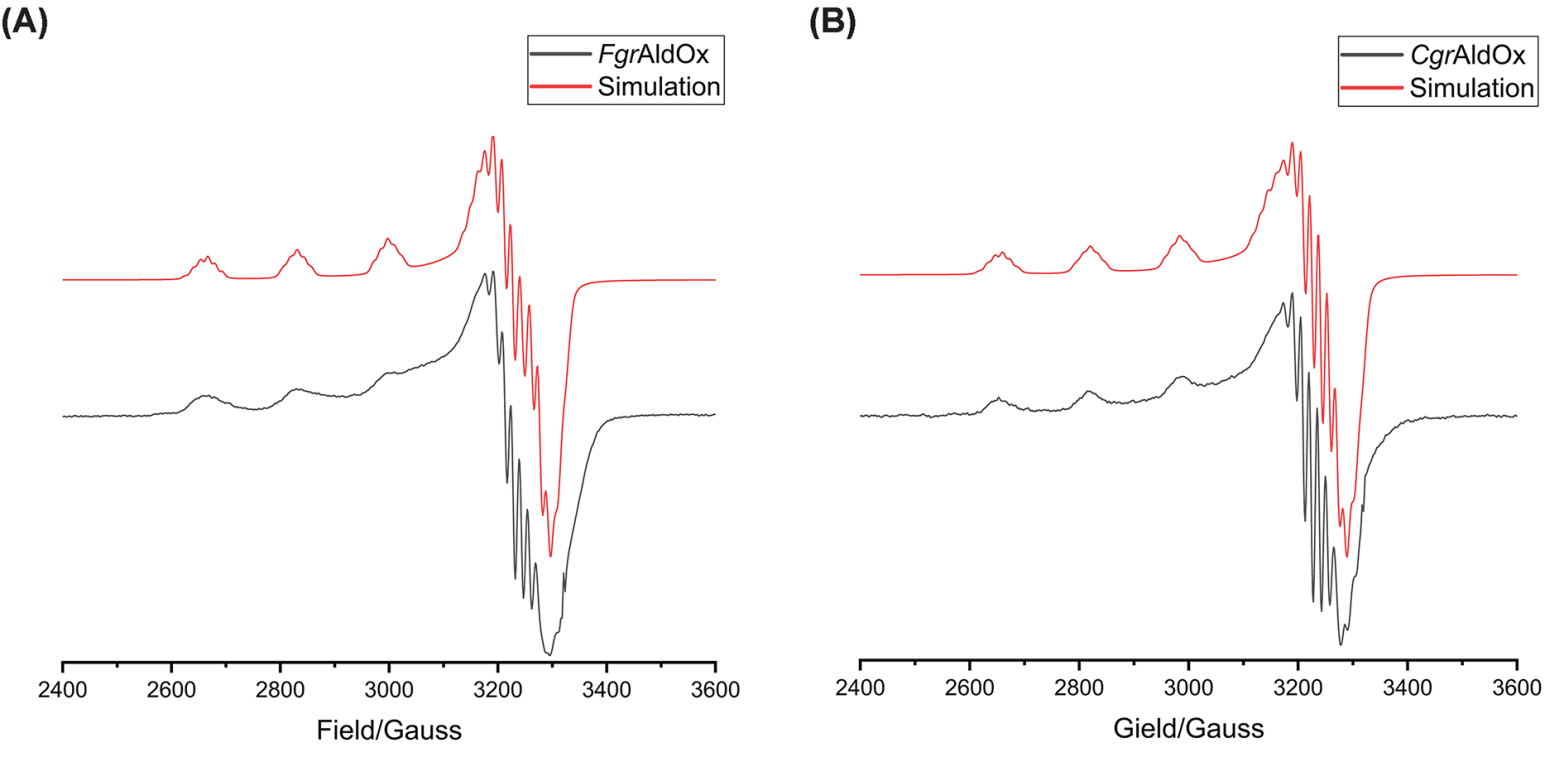
EPR spectra (**A**) *Fgr*AldOx. (**B**) *Cgr*AldOx. Spectra of as-isolated enzyme (black) and the simulated spectra (red).

Spectra of *Fgr*AldOx and *Cgr*AldOx (Figure 2) in the EPR-active, semi-reduced form (Cu^2+^, neutral Tyr-Cys ligand) are similar to those of other CROs, including *Fgr*GalOx [2], *Cgr*AlcOx [32], and *Cgr*AAO [34] of AA5_2, and *Mt*Glox [42] of AA5_1. The copper site geometry exhibits characteristics (*g_z_* = 2.28–2.29, *g_x_* = 2.05, *g_y_* = 2.06), which are typical for axial coordination, i.e., *g_z_* ≠ *g_x_* = *g_y_* [58]. This is in agreement with a distorted square pyramidal arrangement of ligands, comprising two histidines, the phenolic oxygen of the cross-linked Cys-Tyr residue, an axial tyrosine, and an external ligand, as observed *in crystallo* for AA5_2 CROs [12,32,34,45] and *Fgr*AldOx (*vide infra*). The spectra also show defined superhyperfine coupling, which could be satisfactorily simulated with the addition of two nitrogen atoms representing the histidine side chains. Overall, the spectra indicate that the *g_z_* (2.28–2.29) and *A_z_* (515–525 MHz) values for both *Fgr*AldOx and *Cgr*AldOx are close to those of a Type II copper site within the Peisach and Blumberg classification [59]. The *Fgr*AldOx spectrum shows the presence of a second species, as evidenced by the shoulders trailing the peaks (Figure 2A). This second species has previously been observed in studies of *Fg*rGalOx maturation and can be attributed to the presence of a population of premature enzymes where the Tyr-Cys cross-link was not formed [14,60].

#### X-ray crystallography

**Figure 3:**
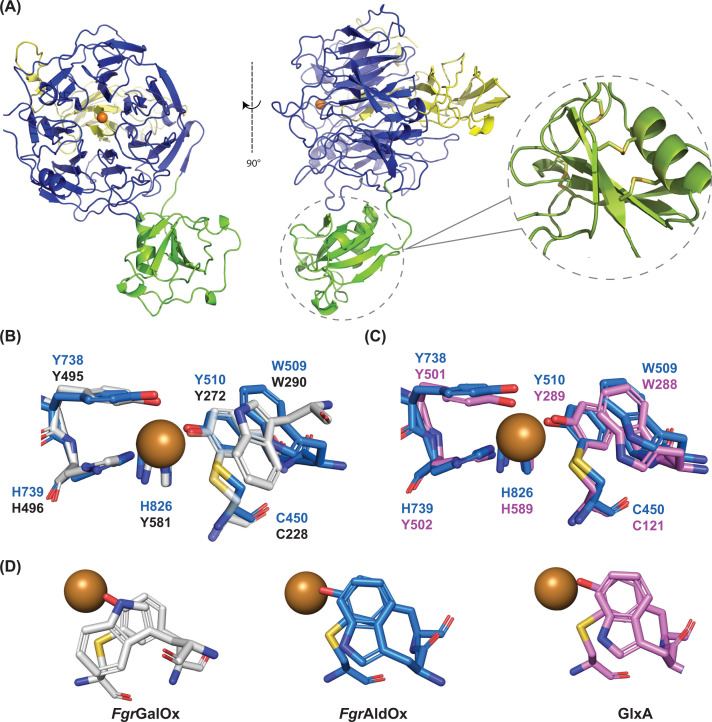
Crystal structure of *Fgr*AldOx (**A**) Front and side view of *Fgr*AldOx. The N-terminal WSC domain is coloured green. The AA5_1 catalytic module comprises the 7-bladed β-propeller domain and C-terminal domain, coloured in blue and yellow, respectively. Inset shows a detailed view of the WSC domain disulphide bridges. (**B**) Overlap of catalytic residues and second coordination sphere tryptophan of *Fgr*AldOx (blue, PDB: 9N3U) and *Fgr*GalOx (grey, 1GOF). (**C**) Overlap of catalytic residues and second coordination sphere tryptophan of *Fgr*AldOx (blue, 9N3U) and GlxA (magenta, 4UNM). Copper ions are coloured in orange. (**D**) Top-down view of the second sphere tryptophan stacked over the Cys-Tyr cofactor in *Fgr*GalOx (grey), *Fgr*AldOx (blue), and GlxA (magenta).

The X-ray crystal structure of *Fgr*AldOx was solved via molecular replacement using an AlphaFold 2 model generated from its primary sequence. As expected for the expression construct, the tertiary structure comprises a single wall stress-responsive component (WSC) domain at the N-terminus and a seven-bladed beta-propeller with a C-terminal immunoglobulin-like domain (Figure 3A). These last two domains form the conserved AA5 catalytic module [12,32,34]. The WSC domain exhibits the characteristic PAN/Apple domain fold, i.e., an antiparallel beta-sheet cradling an alpha-helix. The eight conserved cysteine residues of the WSC domain form four disulfide bridges (Figure 3A). Surface electrostatic potential calculations indicate that *Fgr*AldOx has large negatively charged patches, particularly at the face of the beta propeller motif where the active site is located (Supplementary Figure S13).

The active site comprises a single copper ion coordinated by an unmodified tyrosine (Y738), two histidines (H739, H826), and a modified tyrosine (Y510) covalently linked to a cysteine residue (C450) via the CE2 carbon to form the Cys-Tyr cofactor (Figure 3B,C). The oxygen atom of a solvent water molecule completes the copper coordination. A bioreactor was used for the particular preparation used to obtain this crystal structure, which resulted in reduced copper loading (crystallographic occupancy 0.2). This lower loading was also reflected in a 5-fold lower specific activity of this preparation compared with previous shake-flask production. Previous studies report that copper is required for cofactor biogenesis [60–62]. Interestingly, clear electron density for the Cys-Tyr cross-link was observed in the electron density maps of the structure despite the low copper occupancy. The apparently complete formation of the cross-link under low copper loading may have been due to aerobic, oxidising conditions during recombinant production, crystallisation, or other handling steps.

As introduced above, sequence analysis of *Fgr*AldOx failed to reveal the presence of an obvious aromatic residue in the second coordination sphere, which would be homologous to W290 in *Fgr*GalOx. This aromatic residue is widely found in AA5_2 members and has been implicated in substrate binding and radical stabilisation [52]. The crystal structure strikingly revealed that W509 is instead presented in this position in *Fgr*AldOx, which is provided by a different region of the polypeptide chain than in *Fgr*GalOx. Also remarkable, the orientation of the indole side chain of W509 is flipped (rotated ca. 180 degrees) in comparison with W290 in *Fgr*GalOx (Figure 3B). This flipped orientation has also been observed in the *Streptomyces lividans* (bacterial) glyoxal oxidase, GlxA, in which W288 occupies this second-shell position (Figure 3C). Like W290 in *Fgr*GalOx, W288 is critical for cofactor formation and radical stabilisation in *Sl*GlxA [46]. *Sl*GlxA is not classified as a member of AA5_1 due to low sequence similarity (*vide supra*) yet has a similar activity profile to that of *Fgr*AldOx and other fungal AA5_1 enzymes [45,46]. These results suggest catalytic commonality among diverse fungal and bacterial GlyOx, perhaps mediated, in part, by this alternative second-shell structure.

Unfortunately, crystallisation of *Cgr*AldOx was unsuccessful despite several attempts, yet an AlphaFold3 model indicated a similar orientation of a homologous tryptophan residue (W533; Supplementary Figure S14) in the second coordination sphere. Disentangling the catalytic effect of this alternate tryptophan orientation would be highly challenging, if not intractable experimentally, so we pursued molecular modelling to provide additional insight into aldehyde versus alcohol selectivity of AA5_1 and AA5_2 CROs.

### Molecular modelling *in silico*

We performed long timescale molecular dynamics (MD) simulations (1.8 μs per enzyme) of *Fgr*AldOx (PDB 9N3U, this work) and *Fgr*GalOx (PDB 1GOF, [12]) in their oxidised form (Cu^2+^, radical Tyr-Cys ligand) to investigate how the orientation of the second-shell tryptophan residue affects the structure and dynamics of their active sites. The simulations showed that the second-shell Trp residue (W509 in *Fgr*AldOx and W290 in *Fgr*GalOx) remains quite fixed, with average RMSD values relative to the crystal structures of 0.81 Å and 1.13 Å, respectively, thereby shielding the Tyr-Cys cofactor from the solvent. Notably, the stacking distance between the side chains of Tyr and the second-shell Trp (Y510···W509) remained shorter in *Fgr*AldOx, where the two residues are consecutive, compared with *Fgr*GalOx, where the two residues belong to distinct protein loops. We also note that there appears to be a less complete overlap of this residue with the Tyr-Cys residue in both *Fgr*AldOx and *Sl*GlxA than in *Fgr*GalOx (Figure 3D and Supplementary Figure S16): In *Fgr*GalOx, the 6-membered ring of the Trp is positioned over the thioether and the five-membered ring stacks with the aromatic ring of the Tyr. In contrast, only the 6-membered ring of the Trp stacks with the Tyr, and the five-membered ring is positioned away from the sulphur atom in the Cys-Tyr residue. We anticipate that this distinct difference in overlap may contribute to the measured differences in spin density (*vide infra*).

Another active site difference is the presence of an additional tryptophan residue close to the active site (hereafter named as the ‘third-sphere Trp’) in *Fgr*AldOx that has no homolog in *Fgr*GalOx. Unlike the second-sphere Trp, this third-sphere Trp was very mobile, adopting three main conformations (Supplementary Figure S15). These active site differences between *Fgr*AldOx and *Fgr*GalOx, i.e*.*, the distinct, flipped stacking interaction of the second-sphere Trp and the presence of a third-sphere Trp, could be reasonably expected to affect the electronic structure of the two enzymes, especially the delocalisation of the unpaired electrons in the oxidised form.

**Figure 4:**
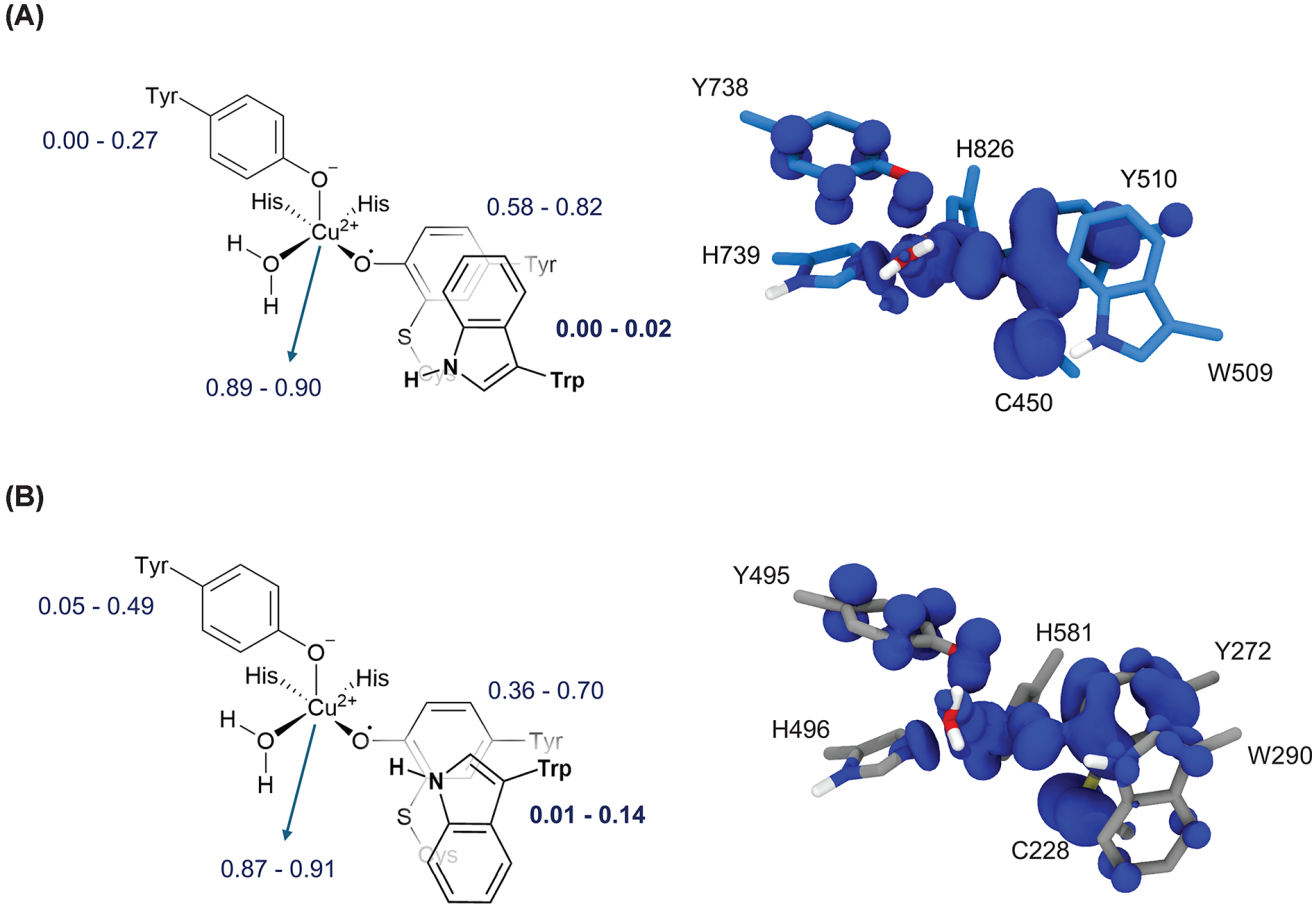
Spin density distribution in the active site of *FgrAldOx* and *Fgr*GalOx (**A**) Left: Schematic picture of the *Fgr*AldOx active site, showing the integrated spin density per residue (small values <0.001 electrons, are not shown), obtained from QM(DFT)/MM calculations in the triplet spin state. The integrated spin density is shown as min–max values obtained from five MD snapshots. Right: Averaged spin density distribution originating from five different MD snapshots. (**B**) Same representations for *Fgr*GalOx.

Hence, we took snapshots from the MD trajectories and computed the spin density distribution of the active site residues using density functional theory (DFT)-based quantum mechanics/molecular mechanics (QM/MM) to gain insight into electronic structure differences between *Fgr*AldOx and *Fgr*GalOx. The spin density was found to fluctuate over time due to the thermal motion of the active site residues. However, clear differences between the two enzymes emerged. Whereas the copper ion holds one unpaired electron in both enzymes, the distribution of the second unpaired electron differs. In *Fgr*AldOx, the second unpaired electron is shared between the Tyr-Cys cofactor and the axial tyrosine, whereas neither the second- nor third-sphere tryptophan residue exhibits significant spin density (Figure 4A). In contrast, the unpaired electron in *Fgr*GalOx is either shared between the Tyr-Cys cofactor and the axial tyrosine or between the Tyr-Cys cofactor and the second-sphere tryptophan (Figure 4B). In other words, only *Fgr*GalOx shows spin density on the second-sphere tryptophan. Notably, a previous study on the *Streptomyces lividans* glyoxal oxidase, GlxA, demonstrated that when the ability to form the cross-link is disrupted by mutation of the key Cys residue, the second unpaired electron migrates to the second-shell Trp [63]. This highlights the central role that the Tyr-Cys moiety plays in electronic stabilisation among distinct CROs.

The absence of spin density on the second-sphere tryptophan in *Fgr*AldOx may result from the flipped orientation of this residue with respect to *Fgr*GalOx and the decreased overlap of the indole ring over the thioether cross-link. It is known that the spin density of an indole radical is higher in the five-membered ring than in the six-membered ring [64]. This explains why only *Fgr*GalOx, in which the five-membered ring overlaps with the cofactor Tyr side chain, exhibits spin density in the second-shell tryptophan. These results suggest that the second-sphere tryptophan plays a more important role in stabilising the radical in *Fgr*GalOx than in *Fgr*AldOx. In *Fgr*AldOx, which oxidises small aldehydes, the unpaired electron appears to require less stabilisation from the second-sphere Trp, consistent with experimental evidence that the radical in aldehyde oxidases is less stable than in galactose oxidases [52]. These differences may contribute to the distinct reactivity and substrate selectivity observed between AA5 subfamilies 1 and 2.

## Conclusion

The characterisation of two orthologous aldehyde oxidases in this work furthers our understanding of the underexplored CROs of AA5 subfamily 1 by providing detailed substrate specificity data, as well as the first experimental three-dimensional structure of a fungal AA5_1 member. In particular, this structure provides a valuable, validated framework for the investigation of the structure-function relationships that modulate the distinct catalytic activities (i.e., aldehyde versus alcohol oxidation) observed between AA5_1 and AA5_2 CROs.

This work also provides a connection between the biochemistry and biology of CROs to further our understanding of their physiological roles. Here, we have shown that *Fgr*AldOx and *Cgr*AldOx oxidise d-glyceraldehyde more efficiently than glyoxal, the substrate that is most commonly associated with AA5_1 CROs. Indeed, in the context of agriculture, identification of the physiological substrates of CROs and the function of specific biochemical pathways in phytopathogenesis may provide avenues for combatting disease and devastating crop losses. Although little is currently known, recent studies continue to unravel the functions of CROs in nature. For instance, although AA5_1 members have long been regarded as H_2_O_2_-producing accessory enzymes for peroxidases during lignin degradation [39,49], studies of glyoxal oxidases from various fungi have since revealed diverse roles in fungal morphology and phytopathogenicity [43,65,66]. In this context, the glyoxal oxidase GlxA from the Gram-positive bacterium *Streptomyces lividans* has been directly implicated in cell wall morphogenesis, where it comprises part of a novel two-component cellulose synthase complex [67]. Not least, *Fgr*AldOx, in particular, has been recognised as a specific virulence factor, antibodies against which confer disease resistance [68]. On the other hand, *Colletotrichum* and *Magnaporthe* AA5_2 alcohol oxidases have recently been implicated in phytopathogenicity via partnered action with peroxidases during penetration and early infection of the plant host [69,70]. Much clearly remains to be discovered regarding the functions of CROs. We envision that further research into the AA5 family using combined biochemical, structural, computational, and biological approaches will advance both fundamental understanding and applications of these enzymes in diverse areas, from biocatalysis to agriculture.

## Materials and methods

### Chemicals and enzymes

All substrates and reagents were purchased from commercial sources (Sigma–Aldrich, VWR, or Toronto Research Chemicals). Lyophilised powders of bovine liver catalase (2000–5000 units/mg, Sigma) and horseradish peroxidase (Rz >3.0, BioBasic Canada Inc.) were used as received. All substrate stocks were prepared with Ultrapure water (18.2 MΩ cm), unless otherwise stated.

### Sequence analysis and bioinformatics

All multiple sequence alignments were aligned with MAFFT. A maximum likelihood (ML) phylogenetic tree containing 150 AA5_1 catalytic modules—omitting any accessory modules along with published AA5_2/AA5 members as an outgroup—was generated from a multiple sequence alignment. Sequence similarity and identity percentages were obtained from BLASTP alignments of sequences containing only the AA5 catalytic module. AlphaFold 2 and 3, accessed via the AlphaFold server at https://golgi.sandbox.google.com/, were used to generate structural homology models of AA5 enzymes with a copper ion [71,72].

### DNA cloning and recombinant strain production

cDNA encoding full-length (WSC-WSC-WSC-AA5_1) and truncated (WSC-AA5_1 and AA5_1) constructs of *Fgr*AldOx and *Cgr*AldOx were ordered for commercial synthesis (GeneWiz, IDT). Full-length *Fgr*AldOx and *Cgr*AldOx were unable to be synthesised; thus, only the truncated gene constructs were obtained. These were cloned into the vector pPICZαA using the EcoRI and XbaI restriction sites, flush with the sequence encoding the *Saccharomyces cerevisiae* α-factor signal peptide. Recombinant plasmids were transformed into chemically competent *Escherichia coli* DH5α by heat shock. To produce the recombinant strains, 5 μg of plasmids containing target sequences were linearised with PmeI and transformed into *Komagataella phaffii* (syn. *Pichia pastoris*) X33 via electroporation. Transformed *K. phaffii* were spread on YPD agar plates containing 100 or 500 μg ml^−1^ of Zeocin. Zeocin-resistant transformants were isolated from plates (EasySelect Pichia System, Invitrogen).

### Protein production

Shake flask protein production was performed as previously described [37]. Briefly, single colonies of *K. phaffii* X33 (syn. *Pichia pastoris*) expressing proteins of interest were streaked onto YPD plates (500 μg ml^−1^ Zeocin) and incubated at 30°C in the dark. YPD precultures (5 ml, 500 μg ml^−1^ Zeocin) were inoculated and shaken for 8 h (30°C, 250 rpm), after which 1 l BMGY in 4 l baffled flasks was inoculated with the precultures and grown overnight (30°C, 250 rpm). Once the BMGY cultures reached OD_600_ of 6–12, the cells were harvested by centrifugation (4000 rpm, 20 min, 20°C) and resuspended in 400 ml BMMY media supplemented with 0.5 mM CuSO_4_ and 3% methanol (v/v). The cultures were shaken in 2 l baffled flasks for 3 days (16°C, 250 rpm) and fed 1% (v/v) methanol every 24 h. On day 3, secreted proteins were separated from cells by centrifugation (4500 rpm, 20 min, 4°C). The pH of the supernatant was adjusted to 7.4–8.0 by the addition of NaOH (5 M), filtered through a 0.45 μm membrane, and left to equilibrate for at least 12 h at 4°C until purification.

Bioreactor production of proteins was carried out in a 3 l bioreactor with an ADI 1030 controller (Applikon Biotechnology). BMGY precultures (100 ml) were inoculated with a single colony and shaken overnight (30°C, 250 rpm). Fermentation basal salts (1 l) supplemented with 12% PTM1 trace elements were inoculated with preculture (60 ml), and the glycerol batch phase was carried out for 18 h (30°C, 1000 rpm). pH was maintained at 6.0 with automatic addition of 5N NH_4_OH. Dissolved O_2_ (%DO) levels were maintained at 40%. After the exhaustion of glycerol in the fermentation media, a 50% (v/v) glycerol feed was applied at 35 ml/h/l initial culture for 6 h. When glycerol concentration became the limiting factor for growth as determined by an increase in % DO, the methanol feeding phase was initiated. Methanol, 0.25% (v/v) was maintained by a feed rate of 8 ml/h. The methanol feeding phase was carried out for 2 days, after which the culture was harvested and centrifuged (4000 rpm, 20 min, 4°C). The collected supernatant was adjusted to pH 7.4–8.0, filtered through a 0.45 μm membrane, and left to equilibrate at 4°C until purification.

### Protein purification

Protein purification was carried out as previously described [37]. Briefly, supernatant was passed through a prepacked 5 ml Ni-NTA column, pre-equilibrated with loading buffer (20 mM sodium phosphate, pH 7.4, 500 mM NaCl, 10 mM imidazole). Proteins were eluted in 1 ml fractions with a linear gradient of 0%–100% of elution buffer (20 mM sodium phosphate, pH 7.4, 500 mM NaCl, 500 mM imidazole). Fractions of interest were pooled, concentrated (10,000 MWCO Vivaspin centrifugal concentrator), and loaded onto a G25 buffer exchange column pre-equilibrated with 50 mM sodium phosphate (pH 7.0) and 100 mM NaCl, then eluted in 2 ml fractions. Fractions of interest were pooled and concentrated as described above. Purified proteins were aliquoted, flash-frozen in liquid N_2_, and stored at −70°C. Proteins were analysed by SDS–PAGE using pre-cast 4%–20% (w/v) polyacrylamide gels (BioRad) and visualised by Coomassie blue R-250 staining. Protein concentrations were determined by measuring A_280_ and by using extinction coefficients calculated with the ProtParam tool on the ExPASY server.

### Analytical protein deglycosylation

The presence of N-glycosylation was determined by treatment of purified *Fgr*AldOx and *Cgr*AldOx with N-glycosidase F from *Flavobacterium meningosepticum* (PNGaseF, New England Biolabs) under deglycosylating conditions. Protein (3–10 μg) was added to 10× glycoprotein denaturing buffer and heated (10 min, 100°C). The samples were diluted to 20 μl with Glycobuffer 2 and tergitol-type NP-40 detergent, then incubated at 37°C for 1 h after the addition of 1 μl PNGaseF. Changes in protein mobility were assessed by SDS-PAGE and visualised by Coomassie blue R-250 staining. Molecular weights were estimated with a standard curve of log (MW) versus Rf of the protein ladder (BLUelf).

### Affinity gel electrophoresis

Polysaccharides (laminarin, xylan, galactomannan, shrimp chitin, arabinogalactan, and xyloglucan) were dissolved in MilliQ water at 60°C to a concentration of 10 mg/ml. Dyes and affinity gels (1× running buffer, 1 mg ml^−1^ polysaccharide, 10% acrylamide/bisacrylamide) were prepared as described [73] using 30% acrylamide/bisacrylamide solution at a ratio of 37:5.1 (BioRad). Gels were loaded with 10 μl of 0.1 mg ml^−1^ protein (50 mM sodium phosphate, pH 7.0 buffer; 20% loading dye). Electrophoresis was conducted at 90 V for 2 h, and the resulting protein bands were stained with Coomassie blue R-250.

### Substrate screen

Activity was surveyed on a panel of substrates (Supplementary Table S3) using the horseradish peroxidase–2,2’-azinobis(3-ethylbenzthiazoline-6-sulfonic acid) (HRP-ABTS) coupled assay in a reaction volume of 200 μl (50 mM sodium phosphate buffer pH 7.0, 0.25 mg ml^−1^ ABTS, and 0.1 mg ml^−1^ HRP) in a 96-well plate at room temperature (ca. 21°C). Absorbance at 415 nm was measured on a BioTek Epoch microplate spectrophotometer (Agilent). All aldehyde, aryl alcohol, and general alcohol substrates were screened at 10 mM. Carbohydrates and glycerol were screened at 300 mM. Attempts to measure activity on glycolaldehyde were precluded by a high background reaction with HRP-ABTS in two independent commercial lots.

### pH activity profile

Enzyme activity across a range of pH values was determined as described [37] using citrate phosphate (pH 5.0–6.0), sodium phosphate (pH 6.0–8.5), and glycine-NaOH (pH 8.0–9.5) buffers. Enzyme activity was measured using the HRP-ABTS coupled assay (as detailed above) with 10 mM methylglyoxal as the substrate.

### Temperature stability

Stock protein was diluted in 50 mM sodium phosphate buffer at the previously determined pH optimum and pre-incubated in a thermocycler set to maintain a temperature gradient between 30 and 50°C. Samples were taken at various time intervals, and enzyme activity on 10 mM methylglyoxal was measured with the HRP-ABTS coupled assay as described above.

### Michaelis–Menten kinetics

Selected substrates in a range of concentrations were used to determine the Michaelis–Menten parameters for all enzymes. Activity was measured using the coupled HRP-ABTS assay with 0.46 mM ABTS and 21 U ml^−1^ HRP in 50 mM sodium phosphate buffer at pH 8.0 and 6.0 at temperatures of 30 and 35°C for *Fgr*AldOx and *Cgr*AldOx, respectively. Measurements were recorded on a Cary 60 UV-VIS spectrophotometer. The Michaelis–Menten equation was fit to the data with OriginPro. In cases where enzyme saturation was not reached, a linear fit of the data was applied with Origin Pro (OriginLab 9.85).

### Product analysis

Oxidation of galactose (110 mM) was performed as described previously [37], with 7 μM (500 μg) purified *Fgr*AldOx in 50 mM sodium phosphate buffer, pH 8.0, for 24 h at 400 rpm, room temperature. Enzymes were removed via ultrafiltration (10 kDa MW cutoff, Vivaspin column), and the filtrate was lyophilised. Lyophilised powder was dissolved in D_2_O and analysed on a Bruker AVANCE 400 Hz spectrometer. Peaks were identified with reference spectra [35]. Percentage conversion was calculated from integration values of relevant peak areas.

Oxidation of methylglyoxal (40 mM) was prepared as previously described [37] with purified *Fgr*AldOx (7 μM, 500 μg) in 50 mM sodium phosphate, pH 8.0, for 24 h at 400 rpm at room temperature. Enzymes were removed by ultrafiltration, and the filtrate was adjusted to pH 3.0 with HCl (1 M). The solution was adjusted to 5 ml, and 1 ml of DNP (3 mg ml^−1^ in acetonitrile) was added, heated until boiling, and left standing at room temperature until methylglyoxal- and pyruvate-hydrazone crystals formed. The crystals were dissolved in acetonitrile and filtered. Samples (10 μl injection) were analysed on an Agilent 1260 Infinity HPLC equipped with a ZORBAX Extend-C 18 column (4.6 × 150 mm, 5 μm). An isocratic method using 35% H_2_O and 65% acetonitrile at a flow rate of 1.4 ml/min was employed with a 10 min stop time and UV detection at 360 nm. Methylglyoxal hydrazone eluted at 7.2 min, and pyruvate hydrazone eluted at 4.7 min. Reference standards of the hydrazones were prepared as above.

Pyruvic acid was measured using an NADH-lactate dehydrogenase coupled enzyme assay kit (K-PYRUV kit, Megazyme). Separate reactions were prepared as above and stirred at room temperature for 0.5–12 h, after which the enzymes were removed by ultrafiltration. Pyruvic acid concentrations were determined by measuring the oxidation of NADH at 340 nm.

### Inductively coupled plasma–mass spectrometry

*Fgr*AldOx and *Cgr*AldOx were treated with 5 mM EDTA for 1 h on ice. EDTA was removed via buffer exchange (Zebra spin column, Thermo Fisher), and enzyme activity pre- and post-EDTA treatment was measured. *Fgr*AldOx (26 μM) and *Cgr*AldOx (9 μM) were digested in an equal volume of concentrated HNO_3_ (67%–70%, environmental grade) at room temperature for 16 h, then heated at 90°C for 2 h and diluted with MilliQ water to a final volume of 10 ml with ^45^Sc (20 ppb) as an internal standard. Inductively coupled plasma–mass spectrometry (ICP-MS) was performed using a quadrupole ICP-MS 7850 (Agilent) equipped with an SPS 4 autosampler. The collision cell was operated in no-gas mode. Calibration was performed with ^63^Cu standards and 20 ppb ^45^Sc as an internal standard (SPEX CertiPrep).

### Electron paramagnetic resonance

Continuous wave (cw) X-band EPR spectra were collected at 150 K for a frozen solution of the target protein with a concentration of ca. 0.15 mM in 50 mM sodium phosphate buffer at pH 6.0. Data were collected using a Bruker micro EMX spectrometer at a frequency of ca. 9.30 GHz, with modulation amplitude of 4 G, modulation frequency of 100 kHz, and microwave power of 2.00 mW. The data were intensity averaged over eight scans, and simulations of the experimental data were performed using the EasySpin 5.2.28 open-source toolbox implemented by MATLAB R2020a software [74].

### Protein crystallisation, X-ray diffraction data collection, and data processing

*Fgr*AldOx was deglycosylated by EndoH treatment and then buffer exchanged into 20 mM HEPES buffer, pH 7.0, and 100 mM NaCl. Crystallisation conditions were screened in sitting-drop 96-well plates pipetted by a mosquito LCP robot (SPT Labtech). Subsequent optimisations were prepared manually in 48-well hanging drop plates. All plates were stored at room temperature. Crystals of *Fgr*AldOx were grown in 48-well hanging drop plates by mixing 2 μl protein (12 mg ml^−1^) with 2 μl reservoir solution (0.2 M lithium sulphate monohydrate, 0.1 M bis-tris buffer pH 5.3, and 29% w/v PEG 3350) and equilibrated against 200 μl solution in the reservoir. The crystals were harvested and flash-frozen in liquid nitrogen without cryoprotectant.

X-ray diffraction data (Supplementary Table S4) was collected at the Canadian Light Source (Saskatoon) on the CMCF-ID beamline [75]. Data sets were auto-processed by the in-house automatic processing pipeline, MXproc. The *Fgr*AldOx (PDB 9N3U) structure was determined by molecular replacement with AlphaFold2 models as search coordinates using the program Phaser [76]. Generated models were refined with Phenix.refine and interspersed with manual building on the WinCoot program [77,78]. The final structure was validated on the validation server of the Protein Data Bank.

### Protein modelling and simulations

The crystallographic structure of *Fgr*AldOx reported in this work (PDB 9N3U) was used as the initial structure for the computational study. The crystallographic structure reported by Ito et al. (PDB 1GOF, at 1.7 Å resolution) was taken as the initial structure for the simulations of *Fgr*GalOx [12]. The acetic acid molecule of the active site was replaced by a water molecule. In both cases, all crystallographic water molecules were retained, and extra water molecules were included to create a 10 Å water box around the protein. Twenty sodium ions and nine chloride ions were required to neutralise the charge for *Fgr*AldOx and *Fgr*GalOx, respectively. The protonation states of aspartate, glutamate, and histidine residues were assigned using the H++ server [79], considering neutral pH. The coordinating Tyr-Cys cofactor and tyrosine were considered as deprotonated.

MD simulations were carried out using the Amber suite of programs and the ff14SB [80] and TIP3P [81] force fields for the protein and water molecules. The deprotonated tyrosine and the Tyr-Cys cofactor were parameterised separately using the Amber force field. The atomic partial charges of the residues and the substrates were calculated at the HF/6-31G* level using Gaussian16 [82]. Lastly, the nonbonded cationic dummy model for Cu^2+^ [83] was used to simulate the copper ion and its environment. The 12–6–4 Lennard–Jones-type nonbonded model was also included in the simulations [84]. The MD simulations were run using Amber20 Molecular Dynamics Package [85].

Energy minimisation was performed in two steps (first solvent molecules, and then the entire system). Afterwards, the system was gradually heated to 300 K (in intervals of 100 K) and then subjected to 500–600 ps of constant pressure to adjust the density of the solution in the NPT ensemble. The production run was extended 0.6 μs for three replicas for both enzymes (1.8 μs for each enzyme). Harmonic restraints (10 kcal mol^–1^ Å^–2^) were added to the coordination bonds of copper during minimisation, heating, and equilibration, and they were subsequently removed before production. The time step of the simulation was 1 fs for the density equilibration and 2 fs for the production run. The SHAKE algorithm was also implemented. Final analysis and clustering were performed with AmberTools [86] and TTClust [87].

Spin densities were calculated with QM/MM using the CP2K software [88]. Energy minimisation calculations were performed on the unliganded structures of *Fgr*AldOx and *Fgr*GalOx (5 MD snapshots per enzyme). The atoms that were treated at the QM level include all residues that coordinate to the copper ion (Y738, H739, H826, Tyr-Cys cofactor, and a water molecule to complete the pyramidal square geometry) and the second-sphere tryptophan (W509 in *Fgr*GalOx). For *Fgr*AldOx, the third-sphere tryptophan (W469) was also included in the QM region. The QM atoms (93 atoms for *Fgr*AldOx and 75 for *Fgr*GalOx) were treated at a DFT/Amber level, with a QM charge of +1 and triplet spin multiplicity. The QM/MM boundary was treated with electrostatic embedding, and broken covalent bonds were capped using the link atom method [89]. Charges of boundary atoms were corrected with AmberTools [81]. Hydrogen atoms with missing Lennard-Jones parameters were corrected using GAFF parameters [90]. The optimisation of the enzyme structures (MD snapshots) was carried out using the Perdew-Burke-Ernzerhof functional (PBE) [91], while the hybrid PBE0 functional [92] was used for single-point calculations to obtain the spin density distribution. Goedecker–Teter–Hutter pseudopotentials [93] were also used, and dispersion corrections were accounted for by using the Grimme D3 type of pair potential (DFT-D3) [94]. Kohn–Sham orbitals were expanded in a plane wave basis using triple-ζ valence polarised basis set functions, with a cutoff of 450 Ry and a kinetic energy cutoff of 70 Ry. Integration of the spin density was performed with a cutoff of 2 Å. Simulation data can be found in the Zenodo repository (doi: 10.5281/zenodo.17641812).

## Supplementary Material

Supplementary Figures S1-S16 and Tables S1-S4

## Data Availability

Coordinates of the X-ray crystal structure of *Fgr*AldOx have been deposited in the Protein Data Bank under the accession 9N3U [95]. All nucleotide sequences, protein sequences, and protein structural information used in this work were extracted from public databases, i.e., GenBank [96], the Protein Data Bank [97], the CAZy database [98], and JGI Mycocosm [99]. All other data generated and analysed during the present study have been included in this manuscript and the associated supplementary information file.

## Open Access

Open access for this article was enabled by the participation of the University of British Columbia in an all-inclusive Read & Publish agreement with Portland Press and the Biochemical Society.

## Abbreviations

AA: Auxiliary Activity Family
AAO: aryl alcohol oxidases, EC 1.1.3.7
AlcOx: alcohol oxidase, EC 1.1.3.13
AldOx: general aldehyde oxidase, EC 1.2.3.-
CAZy: carbohydrate-Active Enzymes classification
CRO: copper radical oxidase
DFT: density functional theory
EPR: electron paramagnetic resonance
GalOx: galactose 6-oxidase EC 1.1.3.9
GlyOx: glyoxal oxidase, EC 1.2.3.15
ICP-MS: Inductively coupled plasma–mass spectrometry
QM/MM: quantum mechanics/molecular mechanics
