# Supplementary material for: Structural enzymology of a Fusarium graminearum aldehyde oxidase reveals a distinct active-site and reactivity versus its paralog galactose oxidase
Source: Biochem J. 2026 Mar 26;483(4):493–509. doi: 10.1042/BCJ20260010 (PMC13094651; doi:10.1042/BCJ20260010)
Supplement: Supplementary Figures S1-S16 and Tables S1-S4 [file BCJ-2026-0010_supp.pdf]

Supplementary Material

Structural enzymology of a *Fusarium graminearum* aldehyde oxidase reveals a distinct active-site and reactivity versus its paralog galactose oxidase

Jessica K Fong[1,2], Laura Mazo[3,4], Alison Nairn[5], Rosa Lorizolla Cordeiro[1], Yann Mathieu[1], Seby Chen[6], Carme Rovira[3,4], Paul Walton[5], Filip Van Petegem[6], Harry Brumer[1,2,6,*]

Affiliations:

[1]Michael Smith Laboratories, University of British Columbia, Vancouver BC, Canada

[2]Department of Chemistry, University of British Columbia, Vancouver BC, Canada

[3]Department of Inorganic and Organic Chemistry and Institute of Theoretical and Computational Chemistry (IQTCUB), University of Barcelona, Barcelona, Spain

[4]Catalan Institution for Research and Advanced Studies (ICREA), Barcelona, Spain

[5]Department of Chemistry, University of York, York, United Kingdom

[6]Department of Biochemistry and Molecular Biology, University of British Columbia, Vancouver BC, Canada

***Corresponding Author:** Harry Brumer, brumer@msl.ubc.ca

Supplementary tables

	***Fgr*AldOx**	*Fgr*GalOx	*Fgr*AAO	***Cgr*AldOx**	*Cgr*AlcOx	*Cgr*AAO	*Cgr*RafOx	*Sl*GlxA
***Fgr*AldOx**		17	18	51	16	19	19	16
*Fgr*GalOx	30		65	18	46	48	48	18
*Fgr*AAO	30	77		17	47	46	46	17
***Cgr*AldOx**	64	31	30		16	19	19	16
*Cgr*AlcOX	29	61	62	28		44	44	18
*Cgr*AAO	31	63	63	33	60		45	19
*Cgr*RafOx	31	57	57	30	56	62		17
*Sl*GlxA	27	30	27	27	28	32	29	

Table S1. Sequence percentage identity and similarity between *F.graminearum* and *C.graminicola* AA5_1 and AA5_2 enzymes.

*Sequence identity and similarity values are highlighted in yellow and orange, respectively. AA5_1 enzymes are in bold font; see main text for literature references.

Query sequence	Subject sequence							
	***Fgr*AldOx**	*Fgr*GalOx	*Fgr*AAO	***Cgr*AldOx**	*Cgr*AlcOx	*Cgr*AAO	*Cgr*RafOx	*Sl*GlxA
***Fgr*AldOx**	100	47	40	100	38	45	48	82
*Fgr*GalOx	50	100	100	65	100	99	99	88
*Fgr*AAO	41	100	100	50	100	99	99	90
***Cgr*AldOx**	100	84	47	100	84	83	44	72
*Cgr*AlcOx	49	100	100	87	100	99	98	97
*Cgr*AAO	61	99	99	52	99	100	100	86
*Cgr*RafOx	51	99	99	71	88	100	100	88
*Sl*GlxA	67	67	65	59	89	82	65	100

Table S2. Percentage query coverage of sequence alignments of AA5 catalytic modules.

*AA5_1 enzymes from this work in bold font.

Table S3. Summary of *Fgr*AldOx and *Cgr*AldOx specific activities on representative panel of substrates.

	**Enzymes and Specific Activities (µmol/min/mg enzyme)**	
**Substrates**	***Fgr*AldOx**	***Cgr*AldOx**
Methylglyoxal	1.7 ± 0.1	12 ± 2
Glyoxal	(3.7 ± 0.5) x 10-2	(8.5 ± 0.6) x 10-2
D-glyceraldehyde	2.1 ± 0.1	27 ± 1
L-glyceraldehyde	(7.3 ± 0.7) x 10-2	(4.0 ± 0.5) x 10-1
Benzaldehyde	(7.3 ± 1.0) x 10-3	(7.0 ± 0.5) x 10-3
Furfural	(7.3 ± 1.0) x 10-3	n.d
Formaldehyde	(1.4 ± 0.1) x 10-1	(5.8 ± 0.1) x 10-1
5-Hydroxymethylfurfural	n.d.	n.d.
Glutaraldehyde	(2.7 ± 0.4) x 10-2	n.d.
Cinnamaldehyde	n.d.	n.d.
Glyoxylic acid	(4.3 ± 1.0) x 10-2	(8.3 ± 0.1) x 10-1
Pyruvic acid	n.d.	n.d.
Galactose	(4.0 ± 0.1) x 10-1	11 ± 1
Melibiose	(2.2 ± 0.7) x 10-2	0.7 ± 0.1
Raffinose	(2.8 ± 0.3) x 10-1	(5.8 ± 0.3) x 10-1
Glycerol	1.8 ± 0.1	6.2 ± 0.3
1,2 – propanediol	(1.4 ± 0.2) x 10-2	(3.7 ± 0.2) x 10-2
1,4 – butanediol	(0.9 ± 0.1) x 10-1	(8.0 ± 0.7) x 10-3
Benzyl alcohol	(1.4 ± 0.5) x 10-2	(5.6 ± 0.2) x 10-2
Hydroxymethyl furfural	(7.3 ± 0.5) x 10-2	n.d.
Hydroxymethyl furan carboxylic acid	(1.8 ± 0.8) x 10-2	n.d.
R-3-Chloro-1,2-propanediol	(16.0 ± 0.4) x 10-1	n.t.
S-3-Chloro-1,2-propanediol	(20.0 ± 0.4) x 10-2	n.t.

*ABTS coupled assay in 50mM sodium phosphate buffer, pH 7.0 at room temperature with 300 mM carbohydrate or polyol, or 10 mM aryl alcohol, aldehyde or carboxylic acid. n.d.= activity not detected at [E] up to 1 mg/mL (15 µM). n.d.=not dected, n.t.= not tested.

Beamline	08ID-1
PDB Code	9N3U
Data collection	
Space group	C 2 2 2_1
Cell dimensions	

Table S4. Data Collection and refinement statistics

a, b, c (Å)	86, 129.98, 134.05
α, β, γ (°)	90, 90, 90
Resolution (Å)	46.66 – 1.28 (1.32 – 1.28)*
R merge	0.083 (1.629)
R meas	0.086 (1.688)
R pim	0.023 (0.439)
$I/\sigma I$	16.7 (1.05)
$CC_{1/2}$	0.999 (0.584)
Completeness (%)	97.6
Redundancy	13.7
Refinement	
Resolution (Å)	1.28
No. reflections	185198
R work/ Rfree	0.14/0.17
No. of atoms	
Protein	4979
Ligand/ion	242
Water	592
B-factors (Å^2)	
Protein	19.62
Ligand/ion	23.02
Water	30.77
r.m.s deviations	
Bond lengths (Å)	0.01
Bond angles (°)	1.28
Ramachandran plot residues	
Ramachandran favoured (%)	96.35
Ramachandran allowed (%)	3.29
Ramachandran outliers (%)	0.16
Rotamer outliers (%)	0.37

*Statistics for highest resolution shell are shown in parentheses

Supplementary Figures

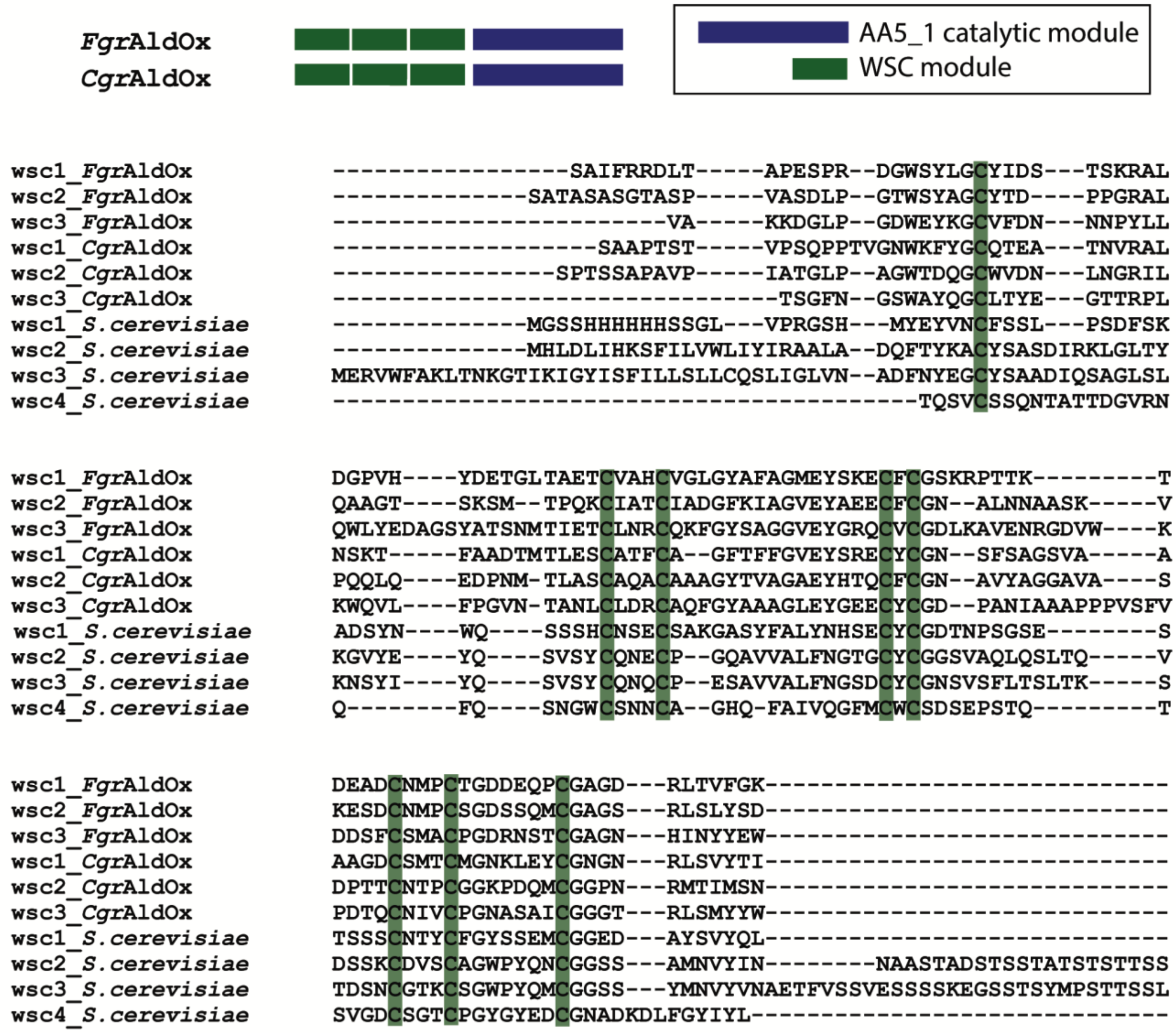

Figure S1. MAFFT sequence alignments of the WSC (wall stress-responsive component) modules from *Fgr*AldOx, *Cgr*AldOx and *Saccharomyces cerevisiae* WSC1-4 proteins. Top: Modularity of *Fgr*AldOx and *Cgr*AldOx. The conserved cysteine residues characteristic to WSC modules are highlighted in green.

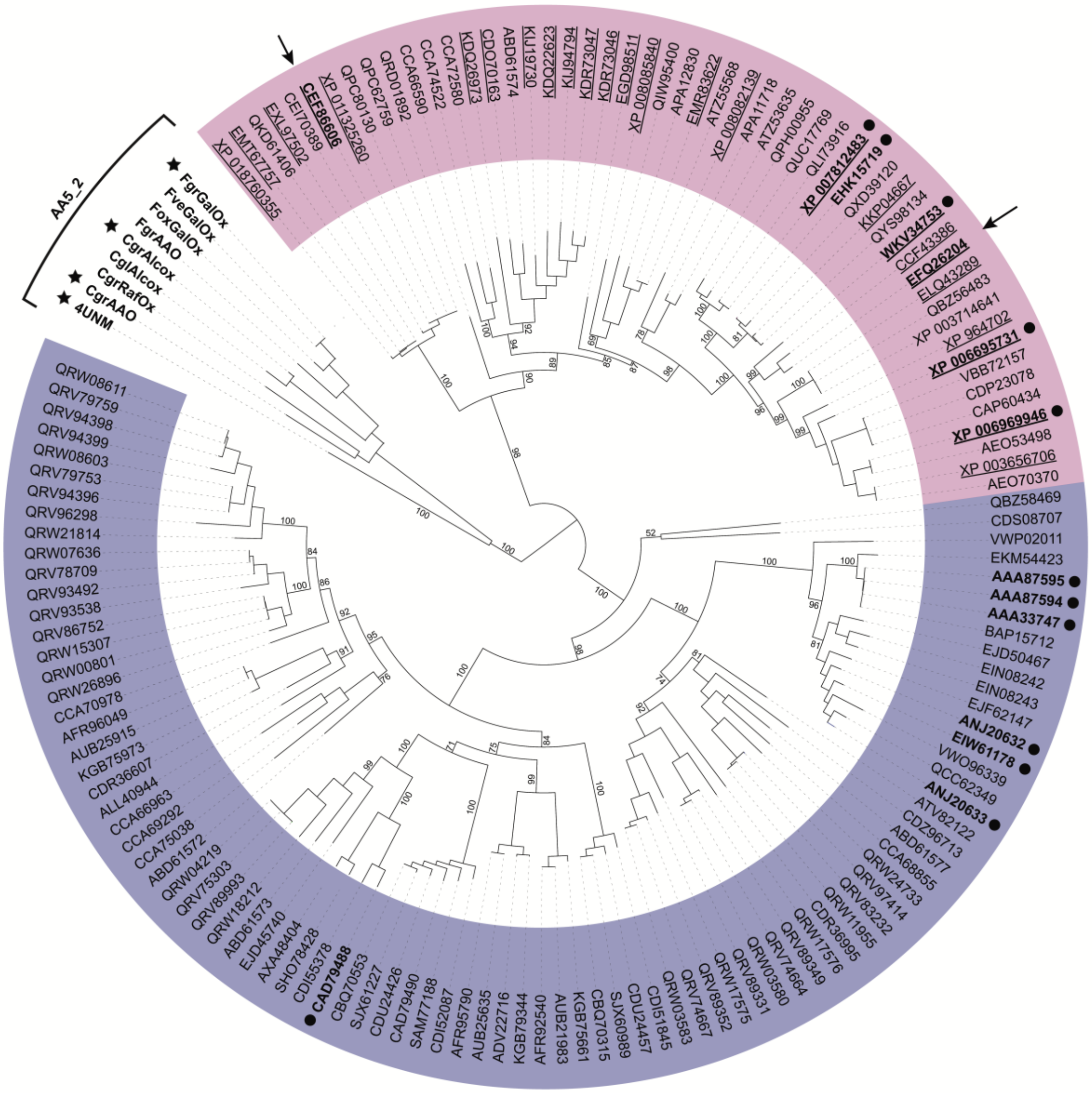

Figure S2. Maximum likelihood phylogeny of AA5_1 members. A curated sequence set of 142 AA5_1 catalytic modules omitting any accessory modules, were used to generate a maximum likelihood phylogeny with characterised AA5_2/AA5 sequences as an outgroup (no background colour). Characterised AA5 members are indicated in bold font. Characterised AA5_1 members are indicated with black circles; *Fgr*AldOx (CEF86606) and *Cgr*AldOx (EFQ26204) from this work are indicated with arrows. Clade of AA5_1 enzymes with and without WSC domains are coloured in pink and blue, respectively. AA5_1 members containing WSC domains are underlined. AA5 members with available crystal structures indicated with black stars.

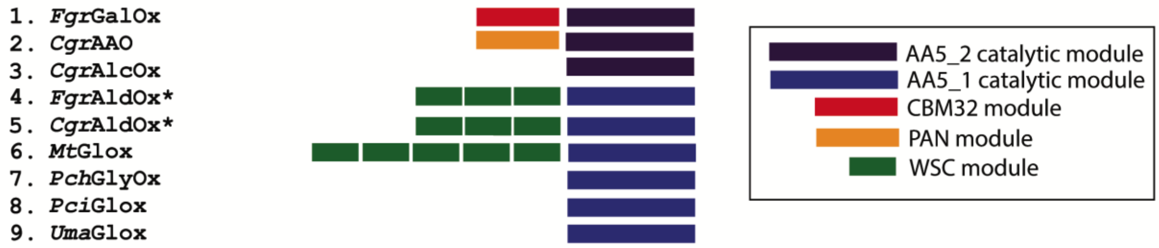


```
                                      228
1. FgrGalOx/213-252   IVSDRTVTVTKHDMFCPGISMDGN--GQIVVTGGNDA------------------KKTSL
2. CgrAAO             AISQRTVTNTHHDMFCPGISQLED--GRILIQGGSDA------------------DTVSI
3. CgrAlcOx           NISDAIVQNTQHDMFCPGTSLDAD--GRIIVTGGSSA------------------AKTSV
4. FgrAldOx*          IYRELALKT---DVFCSASFTLPDKAGRIINIGGWSA------------------ESVYG
5. CgrAldOx*          SWRPMHVKT---DVFCSAGITLPDKAGRQLNLGGWSG------------------DSTYG
6. MtGlox             TFRELHVKT---DVFCAAGVTLPDKAGRQLNIGGWSG------------------DSTYG
7. PchGlyOx           TVRPLNVLT---NSFCASGALLSN--GSMASVGGDPR-GFPGNP---AINP-----GNQA
8. PciGlox            TVRPLSVLT---DSFCASGALLSN--GTMVSMGGTPG-GTGGDV---AAPP-----GNQA
9. UmaGlox            SVTGTAVQT---NTFCASGATLGN--GSWLVAGGNQAVGYGGAAQAQEINPYSDFDGTRA

                                                          272             290
1. FgrGalOx/253-294   YD---SSSDSWIP---------------GPDMQVARGYQSSATMSDGRVFTIGGSWSGGV
2. CgrAAO             YD---PATNEFTR---------------GPNMTLARGYQTSCTLSNGKVFTIGGAYSGER
3. CgrAlcOx           LDFKKGESSPWTP---------------LSNMQISRGYQSSCTTSEGKIFVIGGSFSGAG
4. FgrAldOx*          IRFFTPDSPQGVDNGTNVWEEDYT----QLRLFDPRWYPTALVLSNGSILAMGGESG---
5. CgrAldOx*          VRLYWPDGKPGTP-GTNDWQEDVE----HLRMQDGRWYPSAMIMANGSIFVIGGEEG---
6. MtGlox             TRLYWPDGAPGVP-GTHDWEEDVT----VLKLQSGRWYPSAMVMANGSIMVIGGSIG---
7. PchGlyOx           IRIFEPCASPTGD-GCTVFEDPQT----LHLLED-RWYPSSIRIFDGSLLIVGG------
8. PciGlox            IRIFEPCASPSGD-GCTLFEDPAT----VHLLEE-RWYPSSVRIFDGSLMIIGG------
9. UmaGlox            IRLLEP------N-SQTWIDSPSTTVAQVNMLQQPRWYPGIEVLEDGSVIFIGGAVSGGY

                                                                495
1. FgrGalOx/466-511   ----------STPVFTPEIYVPEQDTFYK-----QNPNSIVRVYHSISLLLPDGRVFNGG
2. CgrAAO             ----------TDGILVAELFNPETREWKQ-----MAPMAVPRNYHSVSILLPDATVFSGG
3. CgrAlcOx           ----------TTPQLTPELFDPATGSFTP-----TTPHTVPRNYHSTALLMADATIWSGG
4. FgrAldOx*          -------GLADNANLNAVMYDPEEPLGQRMTV--LANTTIARLYHSEAVLLSDGKVLVSG
5. CgrAldOx*          -------GLATNPNLNALLYDPRKPIGQRITV--MANTTIARLYHSESITLLDGRVLVSG
6. MtGlox             -------GLAEDPNLNALLYDPTKPLGHRITV--AANTTIARMYHSEAITLLDGRVLISG
7. PchGlyOx           ---------SDHAVLVPSIYTPDAPLGQRISNAGMPSSGIARVYHSSITLTPQGNFLIAG
8. PciGlox            ---------ADHPVLTPSLYTPDAPLGKRISNAGMPTTTIPRMYHSTVTLTQQGNFFIGG
9. UmaGlox            GRTVVTEGLSQDPTYVPVIYDPSKPRGQRLSNANLKPSTIARLYHSSAILLPDGSVMVAG

                                                                      581
1. FgrGalOx/548-591   RTSTQSVKVGG-RITISTD---------------SSISKASLIRYGTATHTVNTDQRRIP
2. CgrAAO             AISADPIKAGA-TLTFTVEG-------------VEGQGTAALIRLGSVTHSVNSDQRRVP
3. CgrAlcox           SLSDTAVRAGA-PITITMQD--------------AGAYTFSMIRVSATTHTVNTDQRRIP
4. FgrAldOx*          ISDRDWTYGES--YTFTL---------TSDLEEGASKLRVSLMASVGATHGVSMGQRTLF
5. CgrAldOx*          LPVTDWAYGST--YTFSL---------GHAPVNGA--IQVSLLGSVSSTHGNSMGARTIF
6. MtGlox             LENRDWAWGET-NITFTL---------GAPAQNGD--ITVTLLGSVSSTHGNSMGARTLM
7. PchGlyOx           NTPEKLAFNKTFTVPISV---------PSTLASPGAKVQVSLMDLGFSSHAFHSSARLVF
8. PciGlox            TMPEKLKFGQKVTVPITI---------PSDLKA--SKVQVALMDLGFSSHAFHSSARLVF
9. UmaGlox            GVPNSVLYGGS---PFNITVNGTFMGDSANAKA--ANTKFAIIRTGFSTHAMNMGQRAVY
```

Figure S3. Domain organisation and MAFFT sequence alignment of select AA5_1 and AA5_2 enzyme catalytic modules. Sequences 1-3 and 4-9 denote AA5_2 and AA5_1 members, respectively. AA5_1 enzymes from this work are indicated with an asterisk. Residue numbers correlate to the amino acid sequence of *Fgr*GalOx. Conserved catalytic amino acid residues are highlighted in green, residues aligning to W290 in *Fgr*GalOx are highlighted in blue. Organism names are abbreviated and in italicised font: *Fgr* (*Fusarium graminearum*), *Cgr* (*Colletotrichum graminicola*), *Pch* (*Phanerochaete chrysosporium*), *Pci* (*Pycnoporus cinnabarina*), *Uma* (*Ustilago maydis*), Mt (*Myceliophthora thermophila*).

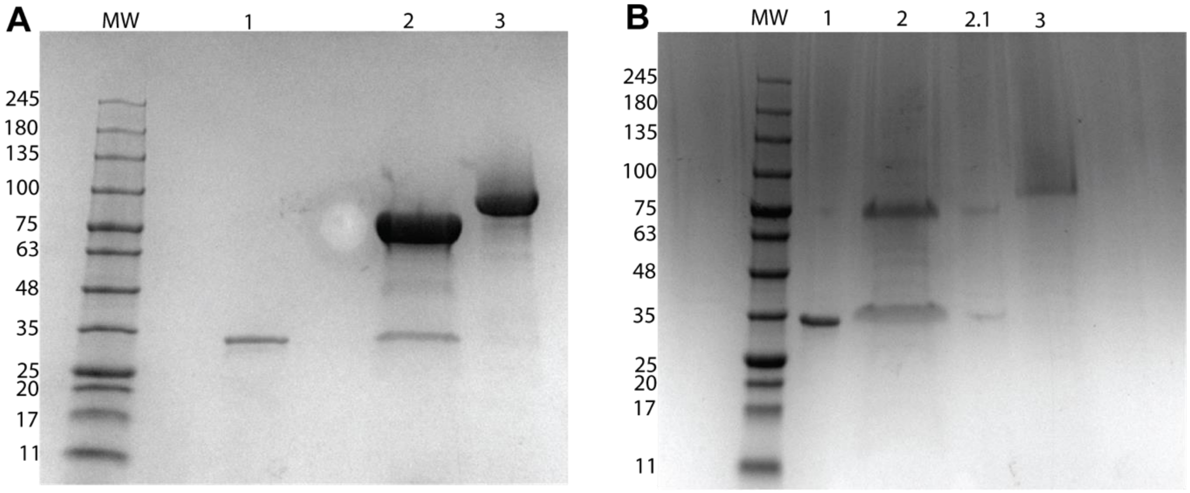

Figure S4. SDS-PAGE of purified *Fgr*AldOx and *Cgr*AldOx, recombinantly produced in *K. pfaffii* (syn. *P. pastoris*) and N-deglycosylation. Aliquots of enzymes N-deglycosylated with PNGaseF and 3 – 10 μg protein was loaded. MW = molecular weight markers, kDa. (A) Lane 1: PNGaseF, 2: PNGaseF + *Fgr*AldOx, 3: *Fgr*AldOx (B) Lane 1: PNGase F, 2: PNGaseF + *Cgr*AldOx, 2.1: sample overflow from lane 2, 3: *Cgr*AldOx. Gels were stained with Coomassie blue.

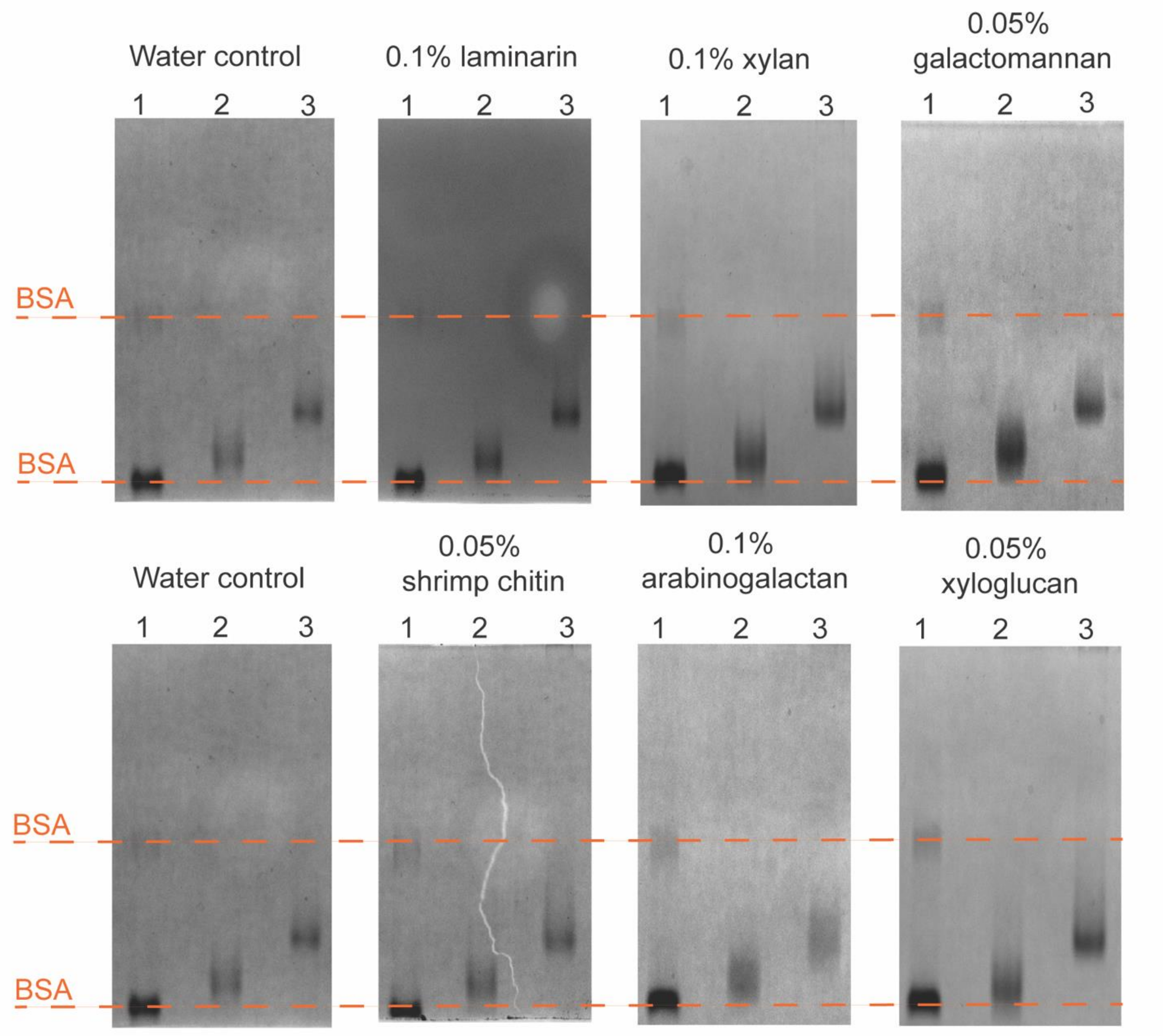

Figure S5. Affinity electrophoresis native-PAGE gels. Gels were loaded with bovine serum albumin, BSA (lane 1), *Fgr*AldOx (lane 2) and *Cgr*AldOx (lane 3). BSA was included as a non-interacting standard and was observed in monomeric and dimeric forms (orange lines). Water control was run without added polysaccharides. Polysaccharide concentrations are listed as w/v.

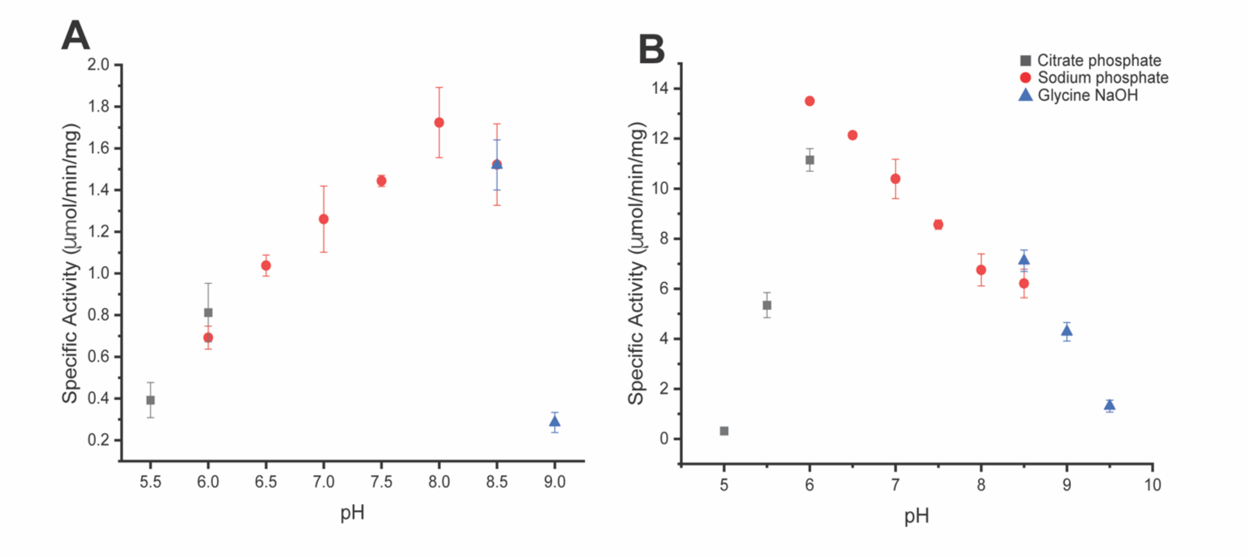

Figure S6. pH-rate profiles. (A) *Fgr*AldOx, (B) *Cgr*AldOx. pH-rate profiles were measured using the HRP-ABTS coupled assay with 10mM methylglyoxal as substrate. Measurements were performed in triplicate at room temperature. Citrate phosphate (black square), sodium phosphate (red circle) and glycine NaOH (blue triangle) buffers were used to cover a range of pH 5.0 -9.5.

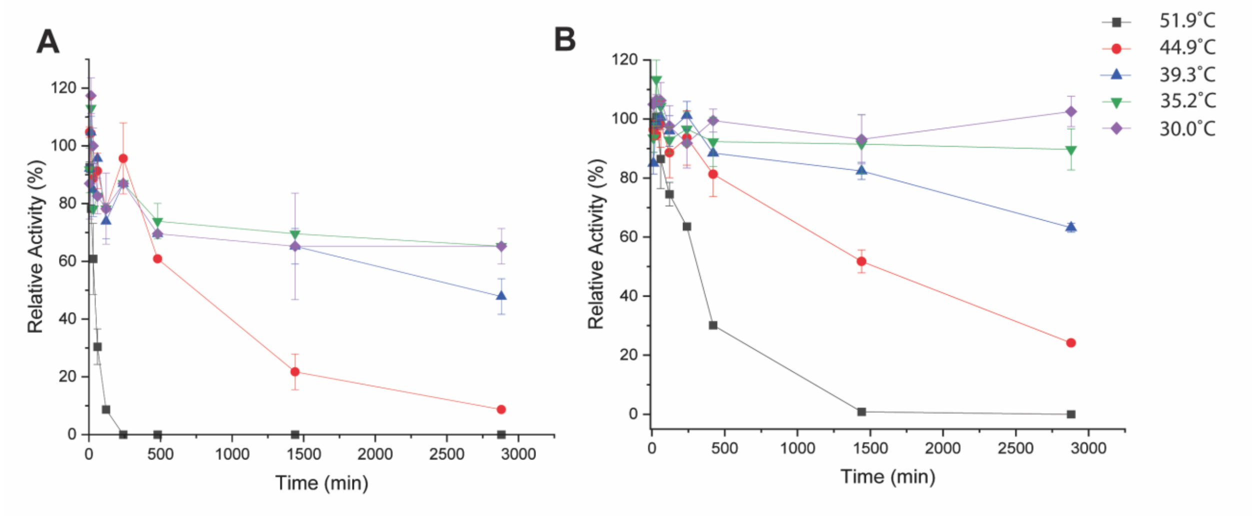

Figure S7. Temperature stability profiles. (A) *Fgr*AldOx, (B) *Cgr*AldOx. Activity was determined using the couple HRP-ABTS assay with 10 mM methylglyoxal as substrate. Reactions were performed in duplicate at room temperature and each enzyme was pre-incubated at each temperature in optimal pH buffer. Temperature was maintained by a gradient thermocycler: 30°C (purple diamond), 35.2°C (green triangle), 39.3°C (blue triangle),44.9°C (red circle) and 51.9°C (black square).

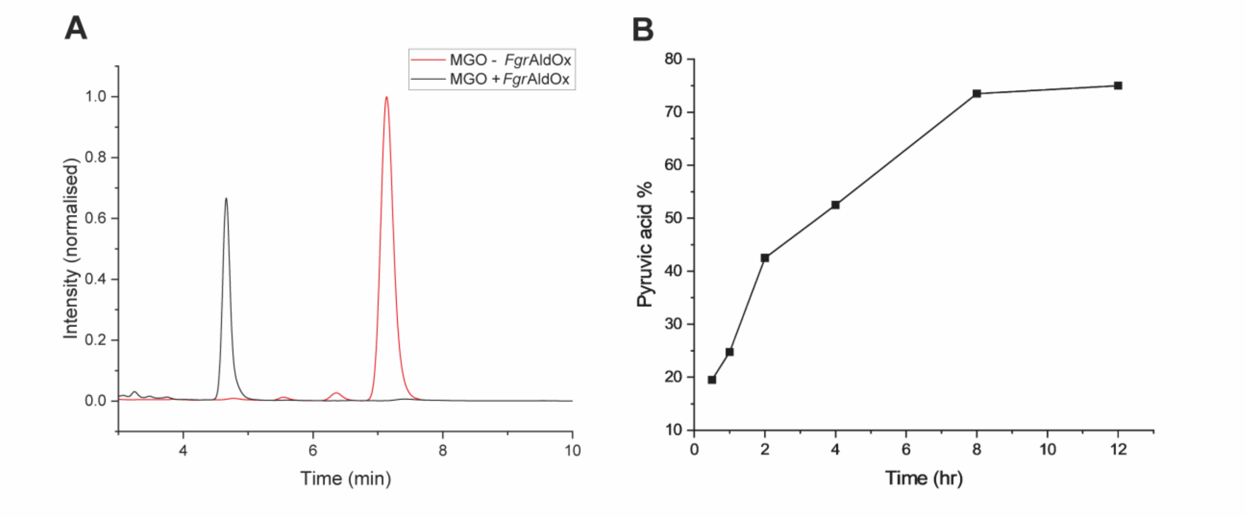

Figure S8. Methylglyoxal (MGO) oxidation by *Fgr*AldOx. (A) HPLC chromatogram of reaction mixtures of 40 mM methylglyoxal with and without 7μM *Fgr*AldOx after derivatization with 2,4-dinitrophenylhydrazine. Peaks corresponding to methylglyoxal-hydrazone and pyruvic acid-hydrazone are represented in red and black, respectively (B) Percentage pyruvic acid after oxidation of 40 mM methylglyoxal at different time point.

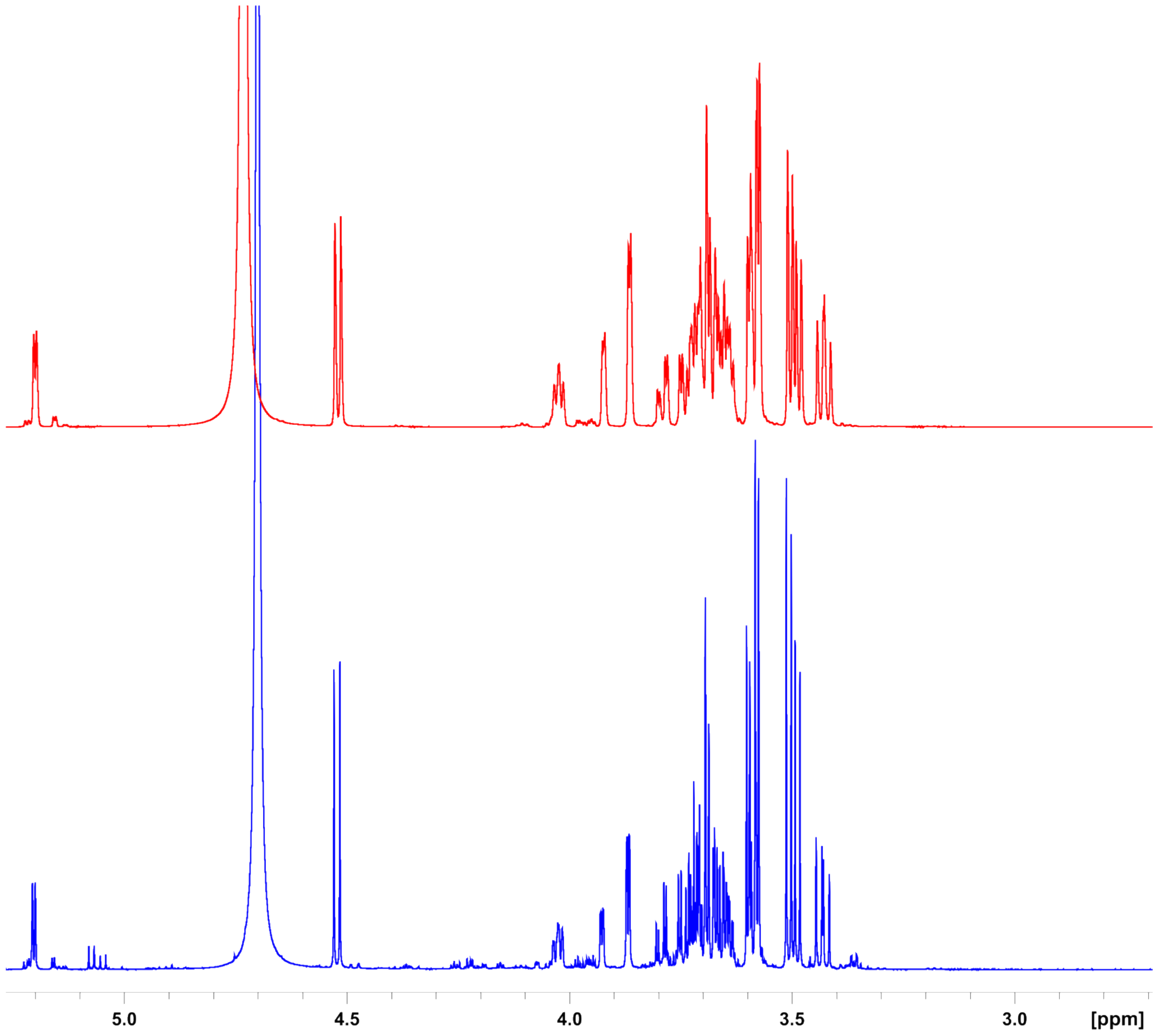

Figure S9. Galactose oxidation by *Fgr*AldOx. ^{1}H NMR spectra for the negative control (red) and reaction with *Fgr*AldOx (blue). A 1:1 ratio of HRP:catalase was added to reactions and were stirred at 400 rpm for 24 hours at ambient temperature. A concentration of 7 μM (500 μg) *of Fgr*AldOx was used in a 1 mL reaction

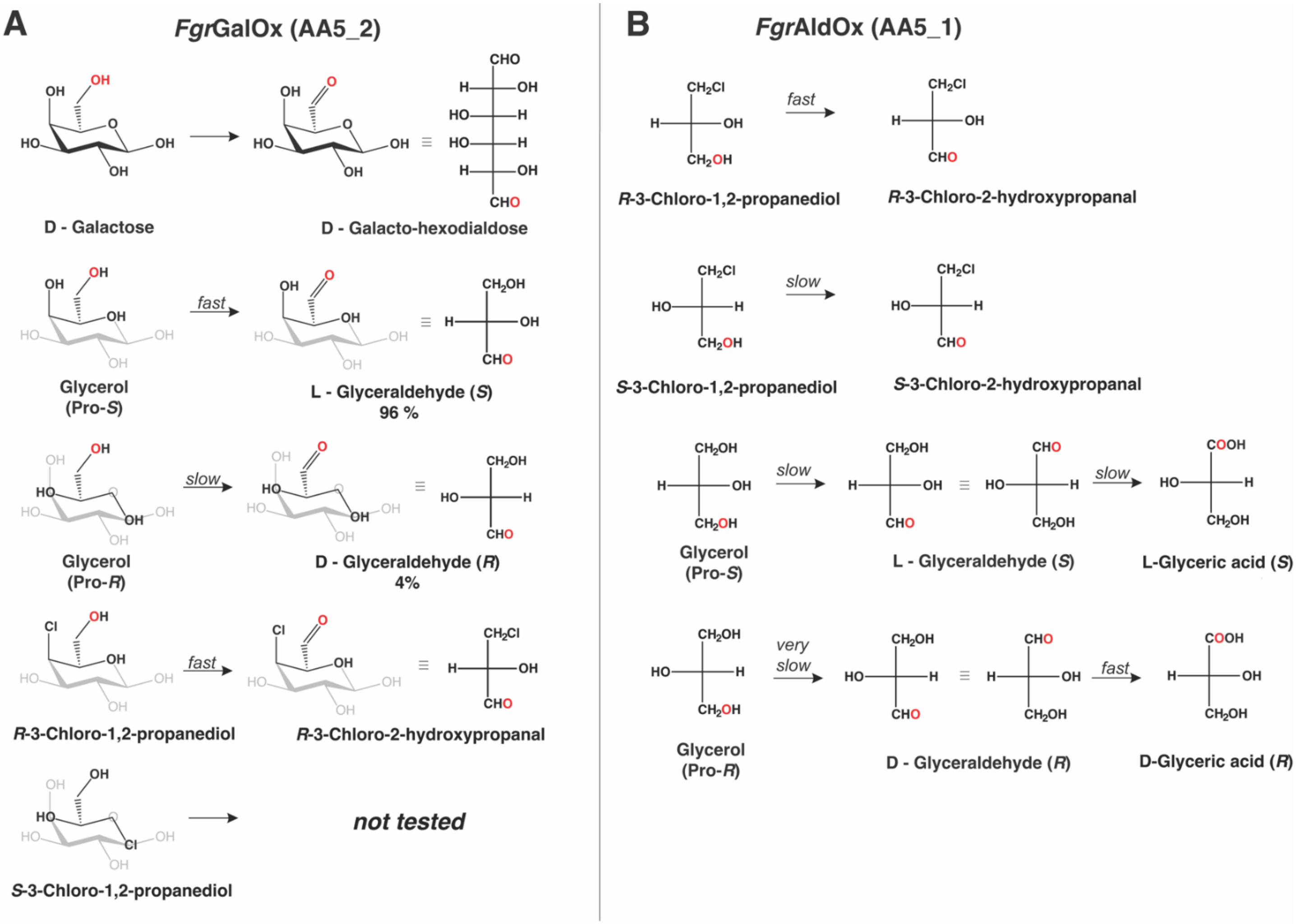

Figure S10. Stereochemistry of glycerol oxidation by *Fgr*AldOx compared to *Fgr*GalOx. (A) Stereochemical analysis of the oxidation of glycerol and chiral analogs by *Fgr*GalOx in reference to galactose conversion (representations and data adapted from Klibanov, *et al. Biochem. Biophys. Res. Comm.* 1982, 108 (2), 804-808, with additional data from Cleveland *et al. Cell. Mol. Life Sci.* 2021, 78 (24), 8187-8208.) (B) Stereochemistry of glycerol and chiral analog oxidation by *Fgr*AldOx.

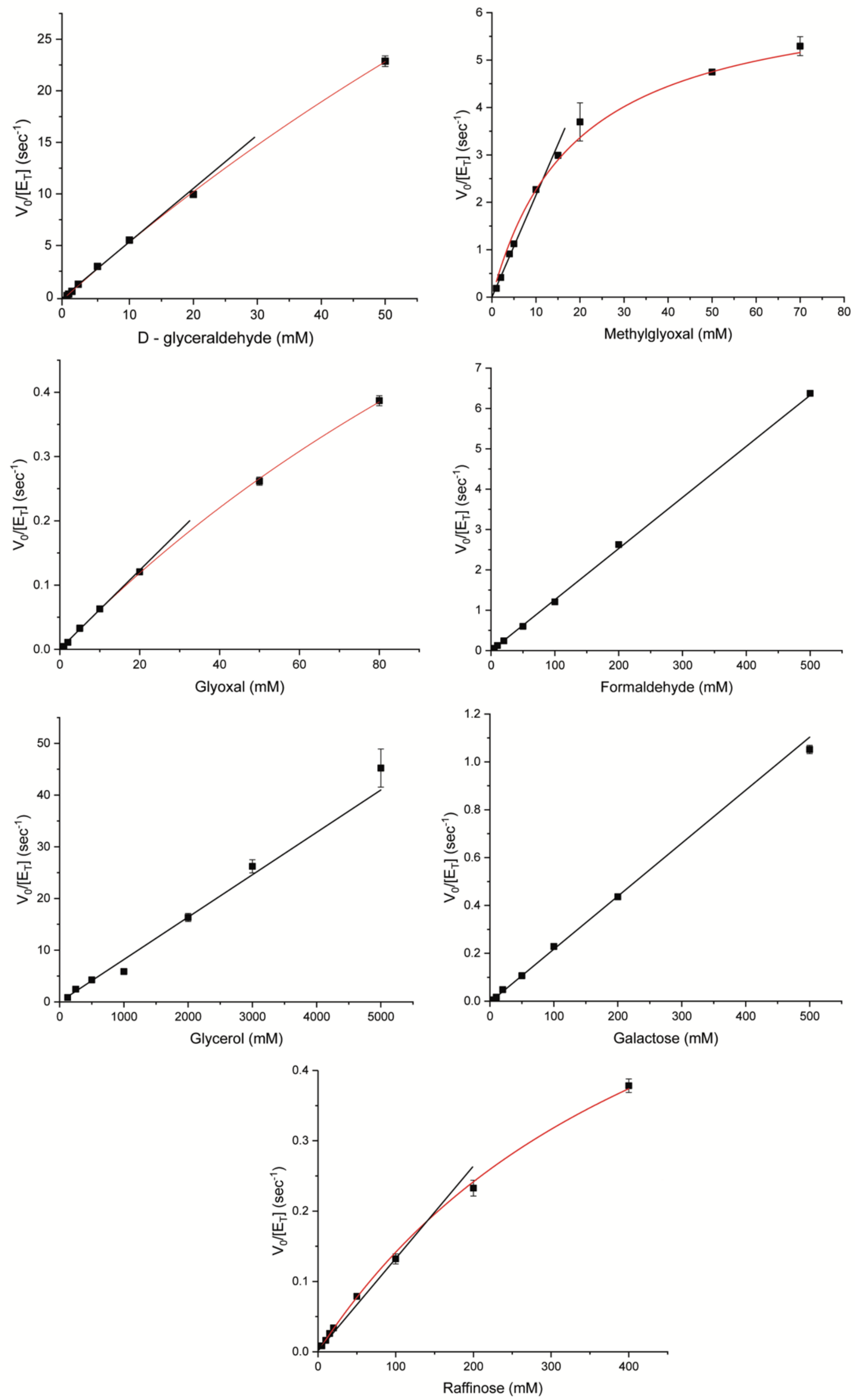

Figure S11. *Fgr*AldOx Michaelis-Menten kinetics. Initial rate values were measured in triplicate at each substrate concentration. Individual k_{cat} and K_M values were calculated by performing a non-linear fitting analysis of the standard Michaelis-Menten equation (red line) using OriginLab 9.85. A linear fit (black line) was also applied to data from reactions which did not reach saturation.

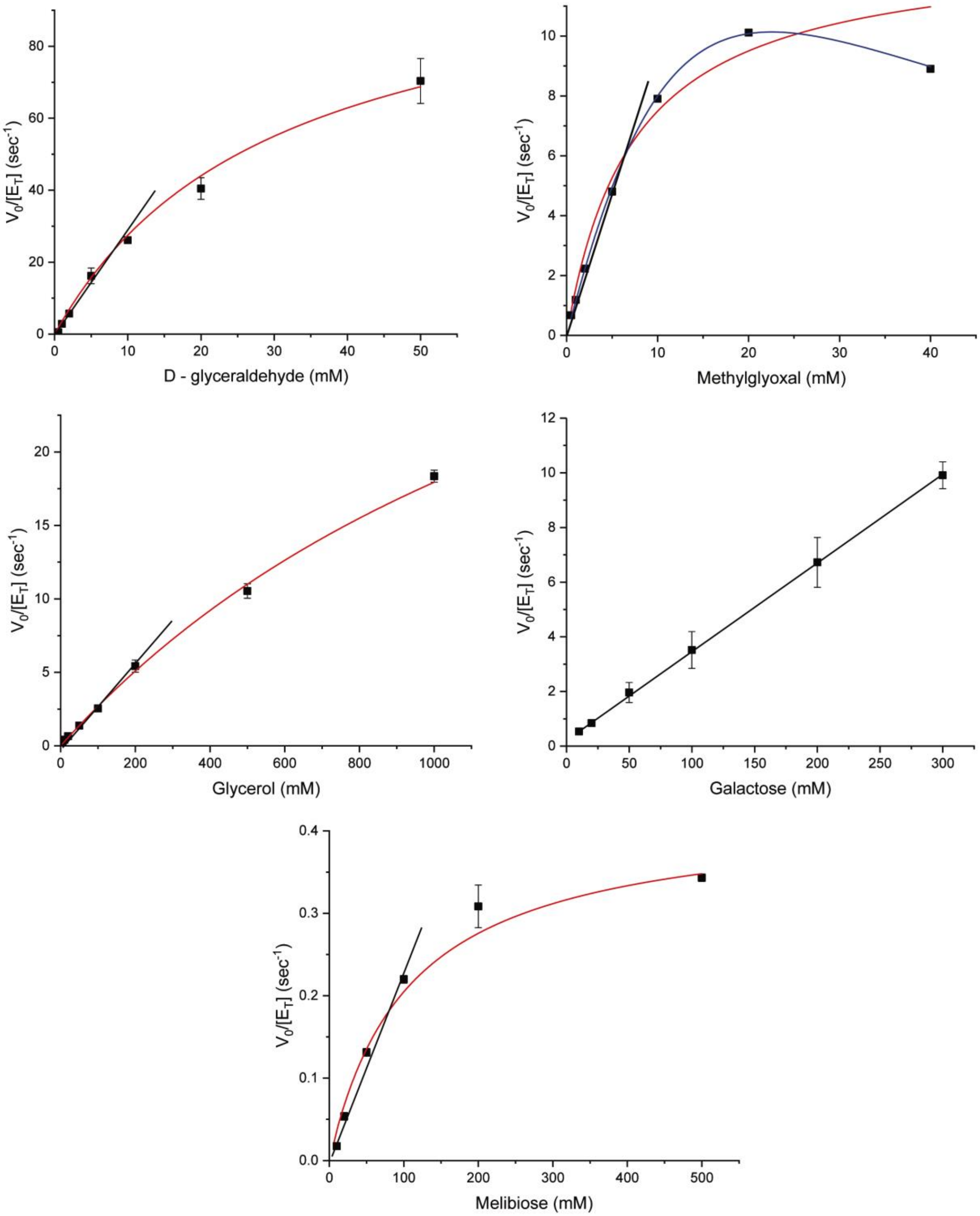

Figure S12. *Cgr*AldOx Michaelis-Menten kinetics. Initial rate values were measured in triplicate at each substrate concentration. Individual k_{cat} and K_M values were calculated by performing a non-linear fitting analysis of the standard Michaelis-Menten equation (red line) using OriginLab 9.85. A linear fit (black line) was also applied to data from reactions which did not reach saturation. *Cgr*AldOx displayed substrate inhibition (blue line) when assayed on methylglyoxal.

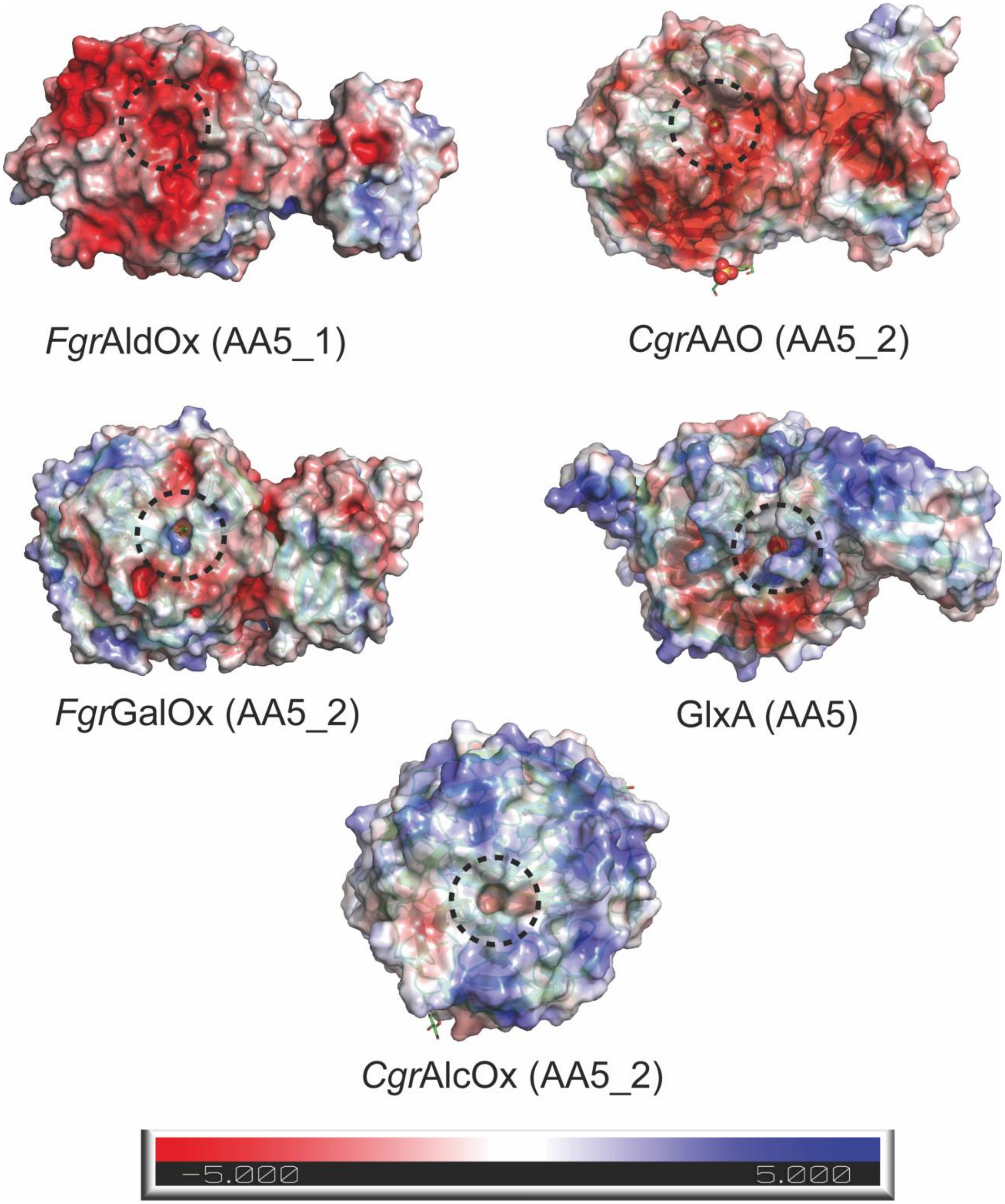

Figure S13. Electrostatic potential visualisation of AA5 enzymes with solved crystal structures. Enzyme names and subfamily affiliation are labeled. Applied charge range for visualisation is indicated by bottom colour key. Active site cavities are circled in black dashed lines.

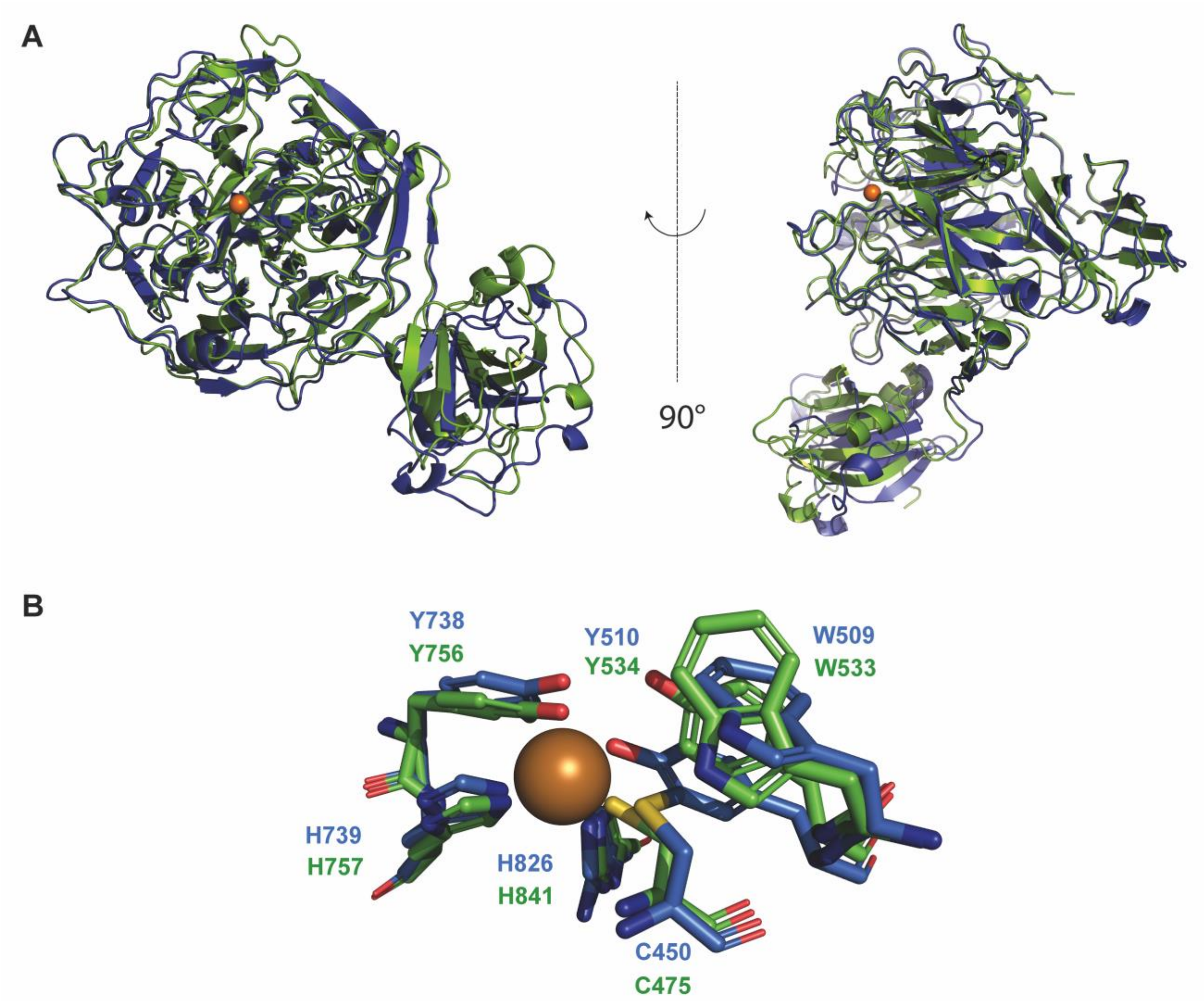

Figure S14. Front and side view of the alphafold 3 model of *Cgr*AldOx aligned to the crystal structure of *Fgr*AldOx. (A) Alphafold 3 model of *Cgr*AldOx (green) and crystal structure of *Fgr*AldOx (blue; PDB 9N3U). (B) Alignment of *Fgr*AldOx (blue) and *Cgr*AldOx (green) catalytic residues.

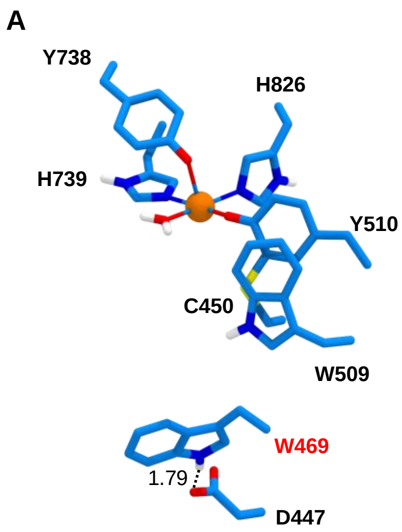

Figure S15. Representative conformations of W509 and W469 in *Fgr*AldOx along the MD simulations. Panel A corresponds to the most predominant conformation (92%), followed by B (5%) and then C (3%).

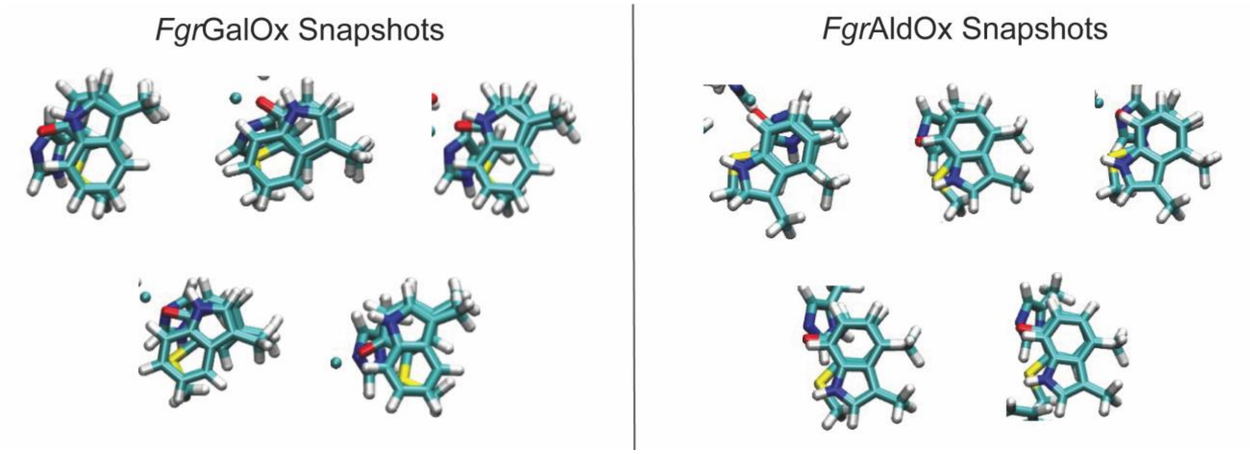

Figure S16. Detailed overlap between the Cys-Tyr crosslink and the second coordination sphere Trp in the five representative MD snapshots used for analysis of the spin density.
